# Glomerular endothelial cell senescence drives age‐related kidney disease through PAI‐1

**DOI:** 10.15252/emmm.202114146

**Published:** 2021-11-02

**Authors:** Camille Cohen, Océane Le Goff, Frédéric Soysouvanh, Florence Vasseur, Marine Tanou, Clément Nguyen, Lucile Amrouche, Julien Le Guen, Oriana Saltel‐Fulero, Tanguy Meunier, Thao Nguyen‐Khoa, Marion Rabant, Dominique Nochy, Christophe Legendre, Gérard Friedlander, Bennett G Childs, Daren J Baker, Bertrand Knebelmann, Dany Anglicheau, Fabien Milliat, Fabiola Terzi

**Affiliations:** ^1^ Université de Paris INSERM U1151 CNRS UMR 8253 Institut Necker Enfants Malades (INEM) Département “Croissance et Signalisation” Paris France; ^2^ Institut de Radioprotection et de Sureté Nucléaire (IRSN) Laboratoire Radiobiologie des Expositions Médicale Fontenay‐aux‐Roses France; ^3^ Service de Néphrologie‐Transplantation Hôpital Necker Enfants Malades AP‐HP centre Université de Paris Paris France; ^4^ Service de Gériatrie Hôpital Européen Georges Pompidou AP‐HP Centre Université de Paris Paris France; ^5^ Service de Biochimie Hôpital Necker Enfants Malades AP‐HP Centre Université de Paris Paris France; ^6^ Service d'Anatomo‐Pathologie AP‐HP Hôpital Necker Enfants Malades AP‐HP Centre Université de Paris Paris France; ^7^ Service d'Anatomo‐Pathologie Hôpital Européen George Pompidou AP‐HP Centre Université de Paris Paris France; ^8^ Department of Pediatrics Mayo Clinic College of Medicine Rochester MN USA; ^9^ Robert and Arlene Kogod Center on Aging Mayo Clinic College of Medicine Rochester MN USA

**Keywords:** aging nephropathy, endothelial–podocyte cross‐talk, kidney transplantation, PAI‐1, senescence, Autophagy & Cell Death, Urogenital System

## Abstract

The mechanisms underlying the development of glomerular lesions during aging are largely unknown. It has been suggested that senescence might play a role, but the pathophysiological link between senescence and lesion development remains unexplained. Here, we uncovered an unexpected role for glomerular endothelial cells during aging. In fact, we discovered a detrimental cross‐talk between senescent endothelial cells and podocytes, through PAI‐1. *In vivo*, selective inactivation of *PAI‐1* in endothelial cells protected glomeruli from lesion development and podocyte loss in aged mice. *In vitro*, blocking PAI‐1 in supernatants from senescent endothelial cells prevented podocyte apoptosis. Consistently, depletion of senescent cells prevented podocyte loss in old *p16 INK‐ATTAC* transgenic mice. Importantly, these experimental findings are relevant to humans. We showed that glomerular PAI‐1 expression was predictive of poor outcomes in transplanted kidneys from elderly donors. In addition, we observed that in elderly patients, urinary PAI‐1 was associated with age‐related chronic kidney disease. Altogether, these results uncover a novel mechanism of kidney disease and identify PAI‐1 as a promising biomarker of kidney dysfunction in allografts from elderly donors.

## Graphical abstract

Kidneys develop lesions with age, and in particular glomerulosclerosis, but the molecular mechanisms involved in the deterioration process are unclear. Here, an unexpected role for glomerular endothelial cells during aging was uncovered.

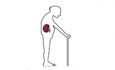

**The paper explained**

**Problem**

As the worldwide population ages, age‐related organ diseases become a real public health burden. Due to the physiological reduction in the number of functional nephrons with age, the prevalence of chronic kidney disease (CKD) dramatically increases in elderly people. CKD is characterized by the inexorable progression toward end‐stage renal disease (ESRD). The survival and quality of life of ESRD patients are very poor. Therefore, there is an urgent need to understand the pathophysiological processes leading to CKD in elderly people in order to develop efficient therapeutic strategies.

**Results**

Here, we identified PAI‐1 as a critical mediator of a detrimental cross‐talk between senescent endothelial cells and podocytes that leads to the development of renal lesions. Deletion of PAI‐1 in endothelial cells or clearance of senescent endothelial cells protected transgenic mice from podocyte loss and glomerulosclerosis with age. More importantly, we demonstrated that these data have a potential great medical impact. In fact, we identified PAI‐1 immunostaining on preimplantation biopsies as an extremely early sensitive biomarker able to predict the suitability of kidneys from elderly donors for transplantation.

**Impact**

As organ shortage for kidney transplantation is a major public health problem, with thousands of people dying each year while waiting for a kidney transplant, the criteria for organ supply have been expanded leading to the consideration of organs from older donors. In this respect, our study provides an important contribution. In fact, we identified PAI‐1 as a novel biomarker, ready to be used in clinical practice and able to predict the survival of kidney allografts from elderly donors.

## Introduction

Chronic kidney disease (CKD) is one of the major public health challenges of the 21st century. Despite efforts from the healthcare community, the survival and quality of life of end‐stage renal disease (ESRD) patients remain severely impaired. CKD is characterized by the progressive decline of renal function to ESRD that occurs once a critical number of nephrons have been lost, irrespective of the cause of renal damage (Hostetter *et al*, 1982). Due to the physiological reduction in functional nephrons with age, the prevalence of CKD dramatically increases in elderly people. Recent studies report that CKD affects 15–38% of people older than 65 years, and rises up to 50% in people older than 85 years (Coresh *et al*, 2007; Hill *et al*, 2016). Hence, there is an urgent need to elucidate the mechanisms underlying the progression of CKD in elderly people in order to develop efficient therapeutic strategies.

Age‐related kidney diseases are characterized by the development of glomerular lesions, such as glomerulosclerosis and podocyte loss (Steffes *et al*, 2001; Appel *et al*, 2009). In living kidney donors, selected because of normal renal function and the absence of any disease susceptible to alter kidney function such as diabetes or hypertension, the percentage of glomerulosclerosis increases with age. In particular, up to 82% of patients older than 70 years display glomerulosclerosis compared with 19% of donors between 18 and 29 years of age (Rule *et al*, 2010). Although this association seems to be robust, the cellular and molecular mechanisms leading to the development of glomerulosclerosis remain unclear. It has been proposed that oxidative stress and ischemia‐induced activation of pro‐inflammatory and pro‐fibrotic pathways may play a role (Valentijn *et al*, 2018). Recently, it has been suggested that cellular senescence may also be detrimental during aging. With the use of a very elegant transgenic mouse model (*p16 INK‐ATTAC*), where senescent cells die by apoptosis as soon as they express p16 upon AP20187 administration, Baker *et al* (2016) have shown that systemic depletion of senescent cells delayed age‐related organ phenotypes, including glomerulosclerosis. It is worth noting that the number of senescent cells increases in kidneys of elderly people (Melk *et al*, 2004). Intriguingly, however, cellular senescence seems to occur mainly in the tubular compartment, while lesions affect almost exclusively the glomerular compartment during aging (Krishnamurthy *et al*, 2004; Melk *et al*, 2004; Baker *et al*, 2016).

Senescence describes a cellular process in which a cell undergoes a permanent cell cycle arrest mediated through the cyclin‐dependent kinase inhibitors, p16 and p21 (Salama *et al*, 2014). Despite the arrest of the cell cycle, senescent cells remain viable and metabolically active and secrete several molecules, known as the senescence‐associated secretory phenotype (SASP). SASP includes pro‐inflammatory cytokines, growth factors, proteases, and extracellular matrix components (Kuilman *et al*, 2008; Salama *et al*, 2014; Watanabe *et al*, 2017). These molecules play a critical role during development and wound healing, but they are also detrimental in pathological processes by favoring a local pro‐fibrotic and pro‐inflammatory environment (Kuilman *et al*, 2008; Childs *et al*, 2015; Watanabe *et al*, 2017). Interestingly, several of these molecules, such as PAI‐1, IL‐6, or TGF‐β, have been shown to play a role in the development of glomerular lesions in several experimental models of CKD (Ding *et al*, 2001; Eddy & Fogo, 2006; Mei & Zheng, 2009). However, since none of the molecules composing the SASP is specific to SASP, it is difficult to determine whether their expression indicates that senescence is involved in this pathological setting or, on the contrary, testifies to the existence of a more general lesional process.

Senescence can be triggered by different stimuli (Kuilman *et al*, 2010). The most classical is the shortening of telomeres observed during aging. Due to the absence of specific repair machinery, telomeres shorten at each cell division. When they reach a critical size, the DNA damage response (DDR) is activated leading to senescence (d'Adda di Fagagna *et al*, 2003; Kuilman *et al*, 2010). By favoring persistent DDR, chemotherapy or X‐rays are also very well‐known inducers of senescence (d'Adda di Fagagna, 2008). Accordingly, irradiation is a widely used technique to induce senescence in experimental *in vivo* and *in vitro* models (Nguyen *et al*, 2018).

Taking all these data together, we hypothesize that senescence might drive age‐related glomerular lesions in a non‐cell‐autonomous way by the secretion of detrimental components of SASP. To investigate this hypothesis, we combined experimental models of aging and genetically modified mice with *in vitro* experiments and human samples. We demonstrated the key role played by endothelial senescence in podocyte loss and subsequent lesion development. Mechanistically, we identified PAI‐1 as a crucial mediator of the interaction between endothelial cells and podocytes. More importantly, we show that these data are relevant to humans and identified PAI‐1 as a promising biomarker for predicting allograft function from elderly donors, as well as kidney function in elderly individuals.

## Results

### Aging nephropathy is associated with glomerular endothelial cell senescence

**Figure 1:**
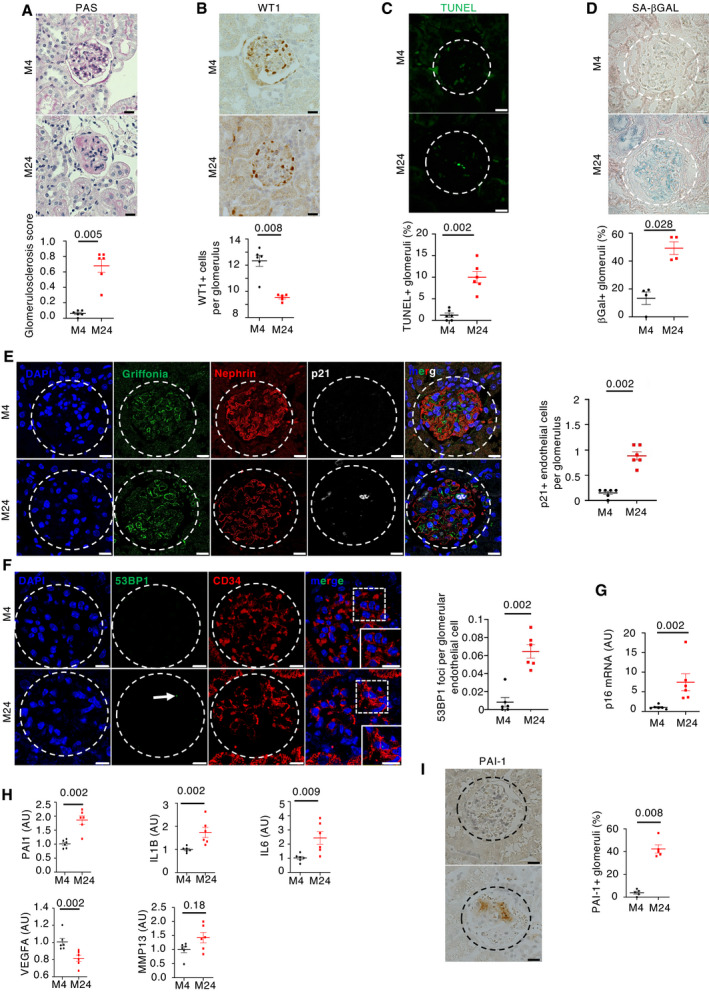
Physiological renal aging is characterized by glomerulosclerosis, podocyte loss, and glomerular endothelial senescence Morphology (PAS staining, upper panels) and quantification of glomerular lesions (lower panel) of kidneys from young and aged mice. Original magnification ×400. Scale bar = 20 μm. *n* = 6 for young and old mice.WT1 immunohistochemistry (upper panels) and quantification of WT1‐positive glomerular cells (lower panel) in kidneys from young and aged mice. Original magnification ×400. Scale bar = 20 μm. *n* = 6 for young and old mice.TUNEL assay (upper panels) and quantification of TUNEL‐positive tubular cells (upper panel) in glomeruli from young and aged mice. Panels are representative images of 6 young and old mice. Scale bar = 20 μm.Senescence‐associated β‐galactosidase staining (upper panels) and quantification of β‐galactosidase‐positive glomeruli (lower panel) in kidneys from young and aged mice. Original magnification ×400. Scale bar = 20 μm. Panels are representative images of four young and old mice.p21/griffonia simplicifolia/nephrin coimmunostaining in kidneys from young and aged mice (left panels) and quantification (right panel) of p21‐positive endothelial cells per glomeruli. Original magnification ×630. Scale bar = 10 μm. Panels are representative images of 6 young and old mice.53BP1/CD34 coimmunostaining in kidneys from young and aged mice (left panels) and quantification (right panels) of 53BP1 foci per glomerular endothelial cell. Original magnification ×630. Scale bar = 10 μm. Panels are representative images of 6 young and old mice.p16 mRNA expression in whole kidney from young and old mice. *n* = 6 for young and old mice.mRNA expression of SASP components in kidneys from young and aged mice. *n* = 6 for young and old mice.PAI‐1 immunohistochemistry (left panels) and quantification of PAI‐1‐positive glomeruli (right panel) in kidneys from young and aged mice. Original magnification ×400. Scale bar = 20 μm. *n* = 5 for young and old mice. Data information: Data are means ± SEM. Statistical analysis: Student's *t*‐test: young vs old mice.

In order to investigate the role of senescence in aging nephropathy, we first compared kidney morphology of 24‐month‐old mice with 4‐month‐old mice. We confirmed that 24‐month‐old mice developed glomerulosclerosis (Fig 1A) together with podocyte loss (Fig 1B). Podocyte loss was associated with an increase in glomerular apoptosis, as revealed by TUNEL assay (Fig 1C).

**Figure EV1:**
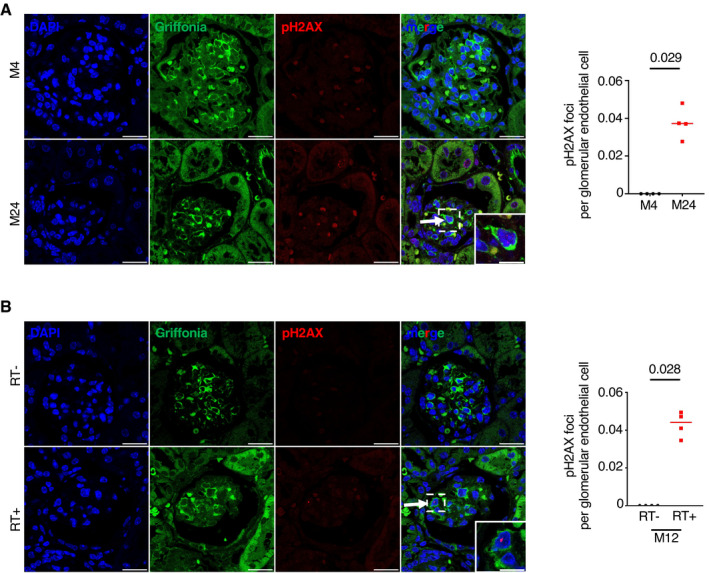
Senescence affects endothelial cells in glomeruli and increases with age A, BpH2AX/griffonia simplicifolia coimmunostaining (left panels) and quantification (right panel) in kidneys from (A) young and aged mice and (B) 12‐month‐old irradiated and non‐irradiated mice. Quantification represents the number of pH2AX foci per glomerular endothelial cell. Original magnification ×630. Scale bar = 10 μm. Panels are representative images of four mice in each group. Data are means ± SEM. Statistical analysis: Student's *t*‐test.

Senescent cells display several characteristics that favor their identification, such as increased lysosomal biosynthesis, cell cycle arrest, or persistence of DNA damage (Kuilman *et al*, 2010). Specific markers can identify each of these events, such as senescence‐associated β‐galactosidase (SA‐βGal), p16 and p21, or p53 binding protein 1 (53BP1). Interestingly, senescent cells were identified mainly in glomeruli of old mice. In fact, SA‐βGal staining was found in 49% of glomeruli in old mice compared with 17% in young mice (Fig 1D). Similarly, immunofluorescence studies using anti‐p21, anti‐53BP1, and anti‐pH2AX antibodies confirmed the presence of senescent cells in glomeruli from old mice (Figs 1E and F, and EV1A). The mRNA level of *p16* was also significantly increased in old mice as compared to young mice (Fig 1G). Glomeruli are composed of different cell types, i.e., endothelial cells, mesangial cells, and podocytes. To characterize which cell type undergoes senescence during aging, we performed coimmunostaining with antibodies directed against p21, a senescence marker, nephrin, a podocyte marker, and griffonia simplicifolia, a lectin that recognizes endothelial cells. Results showed that p21 colocalizes with griffonia simplicifolia, but not with nephrin (Fig 1E). In addition, we observed that the number of p21‐positive cells increased in aged mice as compared to young animals (Fig 1E). To confirm that senescence affects mainly endothelial cells in glomeruli, we performed additional coimmunostaining between the senescence markers, 53BP1 or pH2AX, and the endothelial markers, CD34 or griffonia simplicifolia. We confirmed an increase in endothelial senescence in glomeruli of old mice (Figs 1F and EV1A).

### Glomerular endothelial cell senescence is associated with SASP

**Figure EV2:**
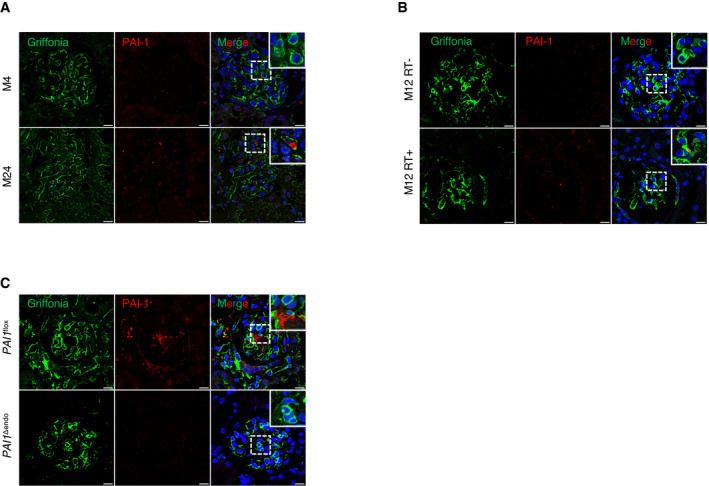
PAI‐1 is expressed in close vicinity to endothelial cells A, BPAI‐1/griffonia simplicifolia coimmunostaining in kidneys from (A) young and aged mice and (B) 12‐month‐old irradiated and non‐irradiated mice.CPAI‐1/griffonia simplicifolia coimmunostaining in kidneys from 22‐month‐old *PAI‐1^flox^* and *PAI‐1^Δendo^* mice. Original magnification ×630. Data information: Scale bar = 10 μm. Panels are representative images of four mice in each group.

The observation that senescence occurred mainly in endothelial cells and apoptosis occurred mainly in podocytes suggests a cross‐talk between these cells. Senescent cells are characterized by the secretion of several molecules, grouped under the name of SASP (Salama *et al*, 2014), suggesting that one of these molecules might drive this cross‐talk. We observed that several components of SASP known to play a potential role in glomerulosclerosis (Wang *et al*, 2017), such as PAI‐1, IL‐1β, IL‐6, or MMP13, were upregulated in kidneys of old mice as compared to young mice (Fig 1H). In contrast, the expression of VEGF‐A was decreased (Fig 1H). Since a number of previous studies showed that the increase in PAI‐1 expression is associated with the development of glomerular lesions in several experimental models of glomerular diseases (Rondeau *et al*, 1990; Yamamoto *et al*, 1993; Grandaliano *et al*, 2000; Lee *et al*, 2001; Hamano *et al*, 2002; Paueksakon *et al*, 2002), we decided to first focus on this paracrine mediator. Immunohistochemical experiments confirmed the increased expression of PAI‐1 protein in old mice, in which 42% of glomeruli were PAI‐1‐positive compared with 4% in young mice (Fig 1I). More importantly, by performing coimmunostaining experiments, we demonstrated that PAI‐1 is expressed in close vicinity to endothelial cells (Fig EV2A). The fact that PAI‐1 is a secreted protein may account for not perfectly overlapping with griffonia simplicifolia staining.

**Figure 2:**
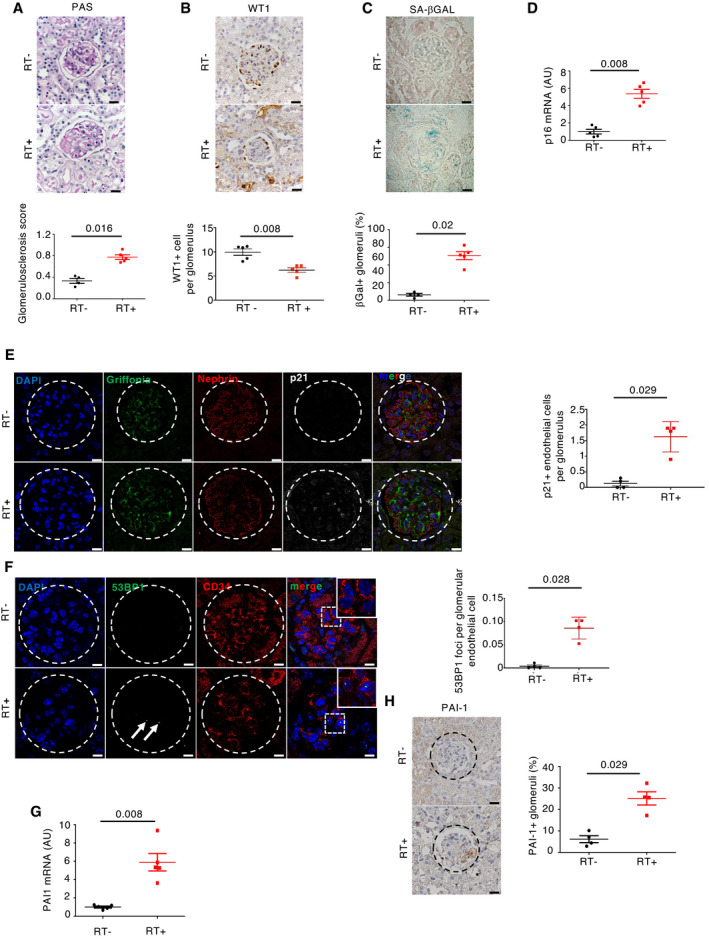
Total body irradiation leads to glomerulosclerosis and glomerular endothelial cell senescence Morphology (PAS staining, upper panels) and quantification of glomerular lesions (lower panel) of kidneys from irradiated (RT^+^) and non‐irradiated (RT^−^) control mice 12 months after irradiation. Original magnification ×400. Scale bar = 20 μm. *n* = 4 and *n* = 5 for RT^+^ and RT^−^, respectively.WT1 immunohistochemistry (upper panels) and quantification of WT1‐positive glomerular cells (lower panel) in kidneys from irradiated (RT^+^) and non‐irradiated (RT^−^) control mice 12 months after irradiation. Original magnification ×400. Scale bar = 20 μm. *n* = 5 for RT^+^ and RT^−^ mice.Senescence‐associated β‐galactosidase staining (upper panels) and quantification of β‐galactosidase‐positive glomeruli (lower panel) in kidneys from irradiated (RT^+^) and non‐irradiated (RT^−^) control mice 12 months after irradiation. Original magnification ×400. Scale bar = 20 μm. *n* = 5 for RT^+^ and RT^−^ mice.p16 mRNA expression in whole kidney from irradiated (RT^+^) and non‐irradiated (RT^−^) control mice 12 months after irradiation. *n* = 5 for RT^+^ and RT^−^ mice.p21/griffonia simplicifolia/nephrin coimmunostaining (left panels) and quantification of endothelial p21‐positive cells (right panel) in glomeruli of kidneys from irradiated (RT^+^) and non‐irradiated (RT^−^) control mice 12 months after irradiation. Original magnification ×630. Scale bar = 10 μm. *n* = 4 for RT^+^ and RT^−^ mice.53BP1/CD34 coimmunostaining (left panels) and quantification of glomerular endothelial cell 53BP1 foci (right panel) in kidneys from irradiated (RT^+^) and non‐irradiated (RT^−^) control mice 12 months after irradiation. Original magnification ×630. Scale bar = 10 μm. *n* = 4 for RT^+^ and RT^−^ mice. Arrows show 53BP1 foci.Relative mRNA expression of PAI‐1 in whole kidneys from irradiated (RT^+^) and non‐irradiated (RT^−^) control mice 12 months after irradiation. *n* = 5 for RT^+^ and RT^−^ mice.PAI‐1 immunohistochemistry (left panels) and quantification of PAI‐1‐positive glomeruli (right panel) in kidneys from irradiated (RT^+^) and non‐irradiated (RT^−^) control mice 12 months after irradiation. Original magnification ×400. *n* = 4 for RT^+^ and RT^−^ mice. Data information: Data are means ± SEM. Statistical analysis: Student's *t*‐test: irradiated vs non‐irradiated mice.

**Figure EV3:**
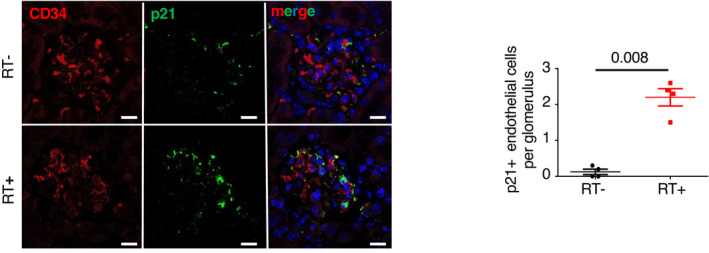
Senescence affects endothelial cells in glomeruli of irradiated mice p21/CD34 coimmunostaining (left panels) and quantification (right panel) in 12‐month‐old irradiated or non‐irradiated mice. Quantification represents the number of p21‐positive glomerular endothelial cells. Original magnification ×630. Scale bar = 10 μm. Panels are representative images of four mice in each group. Data are means ± SEM. Statistical analysis: Student's *t*‐test.

To confirm the possible relationship between senescence and glomerulosclerosis, we studied mice subjected to sublethal total body irradiation, since irradiation is known to induce both senescence and glomerular lesions (Dawson *et al*, 2010; Nguyen *et al*, 2018). As expected, irradiated mice developed glomerulosclerosis (Fig 2A), podocyte loss (Fig 2B), and increased senescence, 12 months after irradiation (Fig 2C and D). Interestingly, p21/CD34, 53BP1/CD34, and pH2AX/griffonia simplicifolia colocalization experiments revealed that senescence affected mainly endothelial cells in glomeruli (Figs 2F, EV1B, and EV3). Similarly, a triple staining with p21/nephrin/griffonia simplicifolia confirmed that p21 is expressed in endothelial cells, but not in podocytes (Fig 2E). PAI‐1 mRNA and protein expressions were also significantly higher in kidneys of irradiated mice as compared to control non‐irradiated ones (Fig 2G and H). Costaining experiments revealed that PAI‐1 is expressed in close vicinity to endothelial cells (Fig EV2B). Together, these data point to endothelial cells as the target of senescence in aging glomeruli and suggest that these cells might determine the fate of podocytes. In this context, PAI‐1 might act as a crucial actor.

### A time course analysis reveals that glomerular senescence and podocyte loss coexist

**Figure 3:**
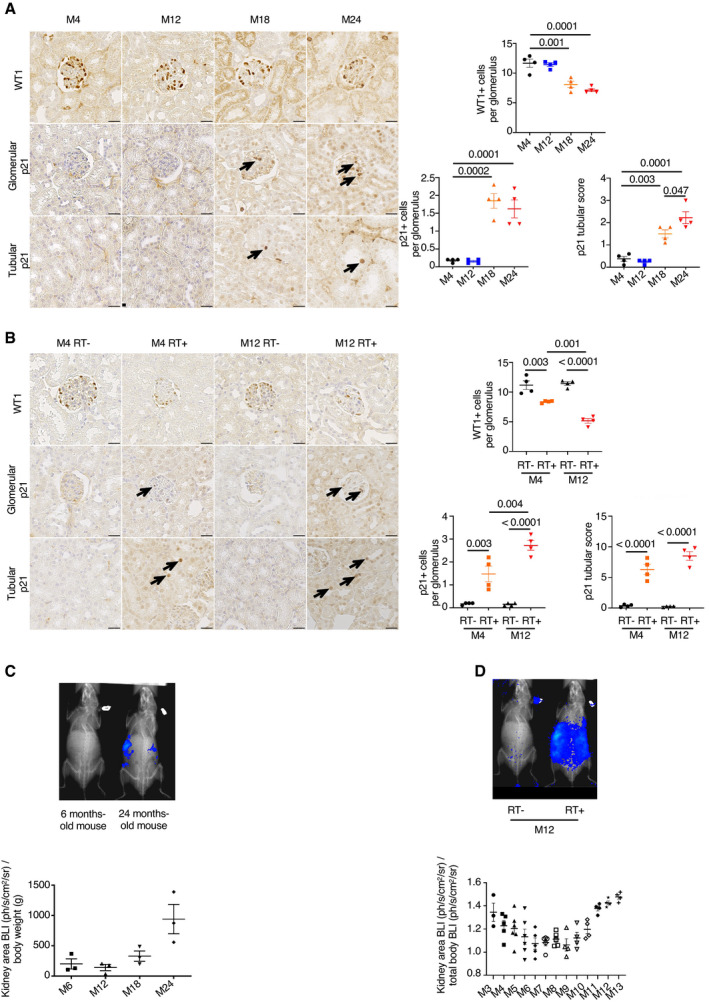
A time course experiment that reveals progressive appearance of senescence and loss of podocytes with age WT1 (left upper panels) and p21 (left middle and lower panels) immunostaining in 4‐, 12‐, 18‐, and 24‐month‐old mice. Quantification of WT1‐positive cells (right upper panels), and glomerular and tubular p21‐positive cells (right lower panels). Original magnification ×400. Scale bar = 20 μm. *n* = 4 for each group. Arrows show positive p21 nuclei, either in glomeruli or in tubules.WT1 (left upper panels) and p21 (left middle and lower panels) immunostaining in 4‐ and 12‐month‐old irradiated (RT^+^) and non‐irradiated (RT^−^) mice. Quantification of WT1‐positive cells (right upper panels), and glomerular and tubular p21‐positive cells (right lower panels). Original magnification ×400. Scale bar = 20 μm. *n* = 4 for each group. Arrows show positive p21 nuclei, either in glomeruli or in tubules.Bioluminescence (BLI, upper panel) and quantification (lower panel) kidney BLI area in young or old mice (upper panel). *n* = 3 mice.Bioluminescence (BLI, upper panel) and quantification (lower panel) kidney BLI area in irradiated (RT^+^) and non‐irradiated (RT^−^) mice over time. *n* = 3–6 mice according to the different time points. Data information: Data are means ± SEM. Statistical analysis: ANOVA followed by the Tukey–Kramer test.

In order to better characterize the kinetics of senescence and podocyte loss in kidney, we performed p21 immunostaining at 4, 12, 18, and 24 months of age. Quantification showed that the number of p21‐positive cells increased from 18 months of age in both tubules and glomeruli (Fig 3A, middle and lower panels). The decrease in the number of WT1‐positive cells paralleled with increased p21 numbers (Fig 3A, upper panels). After irradiation, we observed a similar pattern, with an increase in p21‐positive tubular and glomerular cells as soon as 4 months after irradiation and a decrease in WT1‐positive cells (Fig 3B).

To better trace the appearance of senescent cells in the kidney, we used a model of reporter transgenic mice in which luciferase is expressed under the control of the *p16* promoter (*p16* ^+/^ *^luc^* mice). Bioluminescence confirmed that senescence increased from 18 months of age (Fig 3C). In contrast, in irradiated mice, we observed two peacks of *p16* activation in the kidney area: an early peack, at 3–5 months after irradiation, and a second peack, at 11–13 months (Fig 3D).

### p16‐positive cell clearance prevents podocyte loss and PAI‐1 glomerular overexpression in aged mice

**Figure 4:**
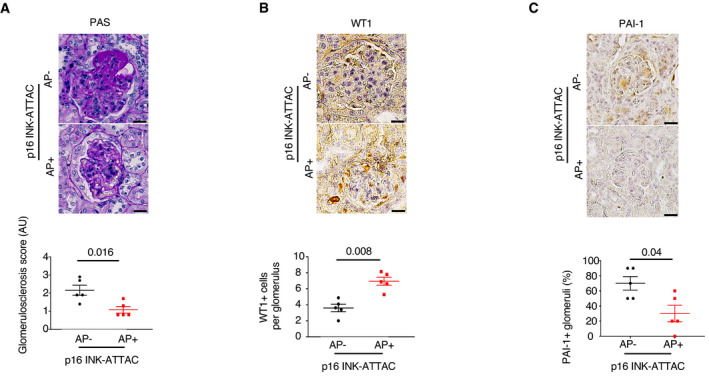
Clearance of senescent cells prevents glomerulosclerosis, podocyte loss, and PAI‐1 overexpression during aging Morphology (PAS staining, upper panels) and quantification of glomerular lesions (lower panel) of kidneys from 28‐month‐old *p16 INK‐ATTAC* mice treated with either vehicle (AP^−^) or AP20187 (AP^+^). Original magnification ×400. Scale bar = 20 μm. *n* = 5.WT1 immunostaining (upper panels) and quantification of WT1‐positive glomerular cells (lower panel) in 28‐month‐old *p16 INK‐ATTAC* mice treated with either vehicle (AP^−^) or AP20187 (AP^+^). Original magnification ×400. Scale bar = 20 μm. *n* = 5.PAI‐1 immunostaining (upper panels) and quantification of PAI‐1‐positive glomeruli (lower panel) in 28‐month‐old p16 INK‐ATTAC mice treated with either vehicle (AP^−^) or AP20187 (AP^+^). Original magnification ×400. Scale bar = 20 μm. *n* = 5. Data information: Data are means ± SEM. Statistical analysis: Student's *t*‐test: AP^+^ vs AP^−^ mice.

To investigate whether senescence is involved in podocyte loss, we studied the *p16 INK‐ATTAC* mouse model. This transgenic model is characterized by the clearance of p16‐positive cells in the presence of AP20187 (Baker *et al*, 2016). We confirmed that the severity of glomerulosclerosis was reduced in aged transgenic *p16 INK‐ATTAC* mice as compared to controls (Fig 4A). More importantly, we demonstrated that the beneficial effect of glomerular senescent cell depletion was associated with an increased number of podocytes at 28 months of age (Fig 4B). Interestingly, glomerular PAI‐1 expression was significantly decreased in these mice (Fig 4C).

### PAI‐1 secretion by senescent glomerular endothelial cells drives age‐related glomerulosclerosis

**Figure 5:**
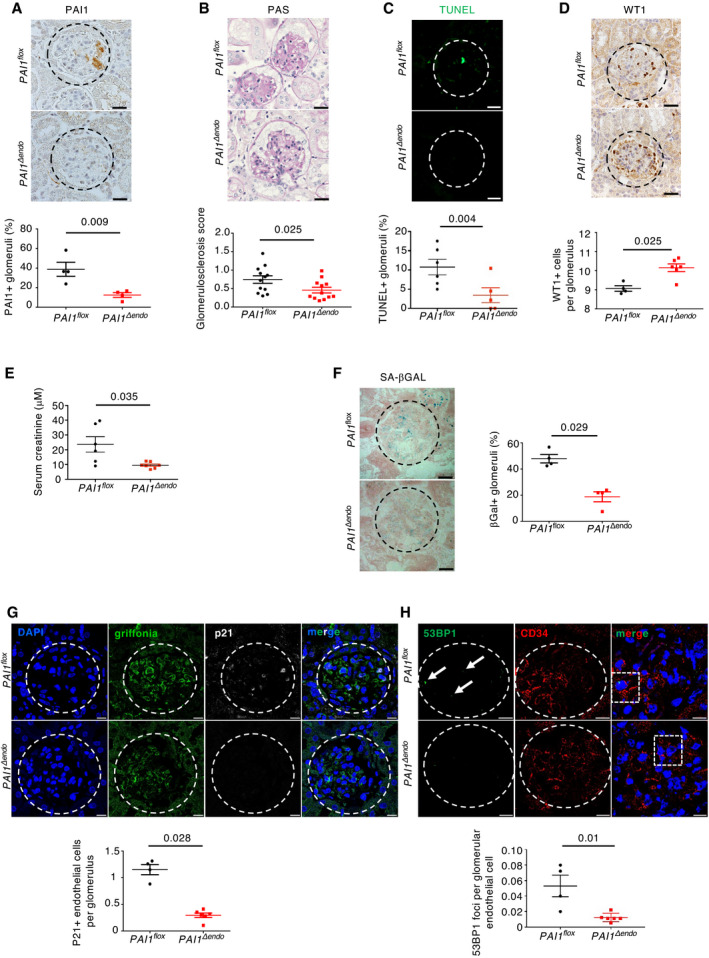
Endothelial‐specific PAI‐1 inactivation prevents age‐associated glomerular lesions PAI‐1 immunohistochemistry (upper panels) and quantification of PAI‐1‐positive glomeruli (lower panel) in kidneys of *PAI‐1^flox^* and *PAI‐1^Δendo^* mice at 22 months of age. Original magnification ×400. Scale bar = 20 μm. *n* = 4 for *PAI‐1^flox^* and *PAI‐1^Δendo^* mice.Morphology (PAS staining, upper panels) and quantification of glomerular lesions (lower panel) of kidneys from *PAI‐1^flox^* and *PAI‐1^Δendo^* mice at 22 months of age. Original magnification ×400. Scale bar = 20 μm. *n* = 12 for *PAI‐1^flox^* and *PAI‐1^Δendo^* mice.TUNEL assay (upper panels) and quantification of TUNEL‐positive glomeruli (lower panel) in kidneys from *PAI‐1^flox^* and *PAI‐1^Δendo^* mice at 22 months of age. Original magnification ×400. Scale bar = 20 μm. *n* = 6 for *PAI‐1^flox^* and *PAI‐1^Δendo^* mice.WT1 immunostaining (upper panels) and quantification of WT1‐positive glomerular cells (lower panel) in kidneys from *PAI‐1^flox^* and *PAI‐1^Δendo^* mice at 22 months of age. Original magnification ×400. Scale bar = 20 μm. *n* = 4 and *n* = 6 for *PAI‐1^flox^* and *PAI‐1^Δendo^* mice, respectively.Serum creatinine measurement in *PAI‐1^flox^* (*n* = 6) and *PAI‐1^Δendo^* (*n* = 7) mice at 22 months of age.Senescence‐associated β‐galactosidase staining (upper panels) and quantification of β‐galactosidase‐positive glomeruli (lower panel) in kidneys from *PAI‐1^flox^* and *PAI‐1^Δendo^* mice at 22 months of age. Original magnification ×400. Scale bar = 20 μm. *n* = 4 for *PAI‐1^flox^* and *PAI‐1^Δendo^* mice.p21/griffonia simplicifolia/nephrin coimmunostaining in kidneys (upper panels) from *PAI‐1^flox^* and *PAI‐1^Δendo^* mice at 22 months of age and quantification (lower panel) of p21‐positive endothelial cells per glomeruli. Original magnification ×630. Scale bar = 10 μm. *n* = 4 and *n* = 6 for *PAI‐1^flox^* and *PAI‐1^Δendo^* mice, respectively.53BP1/CD34 coimmunostaining (upper panels) and quantification of glomerular 53BP1 foci in glomerular endothelial cells from *PAI‐1^flox^* and *PAI‐1^Δendo^* mice at 22 months of age. Original magnification ×630. Scale bar = 10 μm. *n* = 4 and *n* = 6 for *PAI‐1^flox^* and *PAI‐1^Δendo^* mice, respectively. Arrows show 53BP1‐positive foci. Data information: Data are means ± SEM. Statistical analysis: Student's *t*‐test: *PAI‐1^Δendo^* vs *PAI‐1^flox^* mice.

Hence, we next investigated whether PAI‐1 might mediate the detrimental cross‐talk between senescent endothelial cells and podocytes during aging. Toward this goal, we crossed *PAI‐1^flox^* mice with mice expressing a Cre‐recombinase specifically expressed in endothelial cells (*VE‐Cadh‐Cre* mice) in order to generate *PAI‐1^Δendo^* mice. As expected, *PAI‐1^Δendo^* old mice showed a dramatic decrease in PAI‐1 glomerular staining, confirming that the main source of PAI‐1 in glomeruli during aging is the endothelium (Figs 5A and EV2C). Remarkably, the selective deletion of PAI‐1 in the endothelium was able to prevent the development of glomerulosclerosis at 22 months of age (Fig 5B). Similarly, glomerular apoptosis was significantly lower (Fig 5C) and, by consequence, the number of podocytes was higher (Fig 5D) in *PAI‐1^Δendo^* old mice as compared to *PAI‐1^flox^* mice. Additionally, renal function was also significantly improved in *PAI‐1^Δendo^* old mice (Fig 5E). It is worth noting that *PAI‐1^Δendo^* old mice showed less glomerular senescence than *PAI‐1^flox^* old mice (Fig 5F–H), consistent with a previous report showing that PAI‐1 is involved in the maintenance of senescence (Eren *et al*, 2014).

### Endothelial PAI‐1 induces podocyte detachment and apoptosis

**Figure 6:**
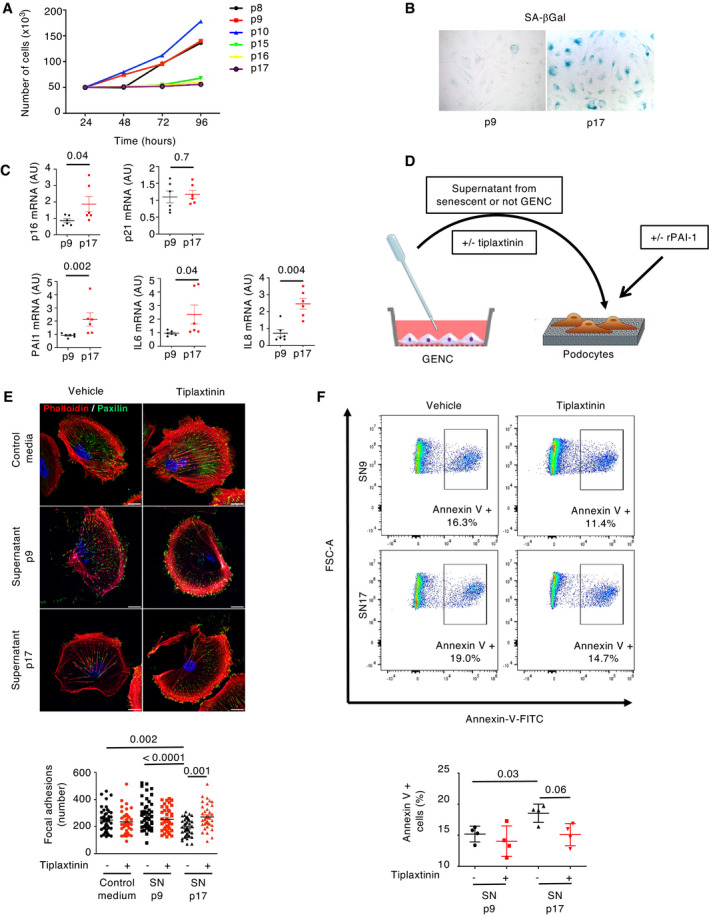
PAI‐1 secretion drives the endothelial cell–podocyte cross‐talk during senescence ATime of cell population doubling of primary glomerular endothelial cells from passage 8 (p8) to passage 17 (p17).BSenescence‐associated β‐galactosidase staining of glomerular endothelial cells (GEnC) at early (p9) or late (p17) passage.CRelative mRNA expression of senescence markers (p16 and p21) and SASP molecules (PAI‐1, IL‐6, IL‐8) in GEnC at early (p9) or late (p17) passage. *n* = 5 independent experiments.DScheme of the experimental protocol used for coculture experiments with glomerular endothelial cells (GEnC) and podocytes.EImmunofluorescence of paxillin (green) and phalloidin (red) in podocytes stimulated with control medium, supernatant (SN) from glomerular endothelial cells from early (p9) or late (p17) passage and treated either with vehicle or with tiplaxtinin, a PAI‐1 inhibitor (upper panel). Quantification of focal adhesions (lower panel) was performed by counting the number of paxillin‐positive spots in 30 cells from three independent experiments. Original magnification ×1,000. Scale bar = 20 μm.FAnnexin‐V‐FITC staining (upper panel) and quantification by FACS (lower panel) of podocytes stimulated with supernatant (SN) from GEnC at early (p9) or late (p17) passage and treated either with vehicle or with tiplaxtinin. *n* = 4 independent experiments. Data information: Data are means ± SEM. Statistical analysis: Student's *t*‐test for (C); ANOVA followed by the Tukey–Kramer test for (E) and (F).

**Figure EV4:**
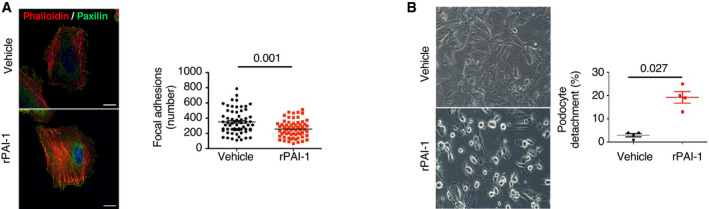
Recombinant PAI‐1 induces podocyte cytoskeleton modifications and detachment Immunofluorescence of paxillin (green) and phalloidin (red) on podocytes stimulated for 30 min with recombinant PAI‐1 at 5 nM. Quantification of focal adhesions (right panel) was performed by counting the number of paxillin‐positive spots in 30 cells from three independent experiments. Original magnification ×1,000. Scale bar = 20 μm.Podocyte morphology (left panels) and quantification of podocyte detachment (right panel) 30 min after stimulation by recombinant PAI‐1 at 5 nM. Original magnification ×400, *n* = 4 independent experiments. Data information: Data are means ± SEM. Statistical analysis: Student's *t*‐test.

To provide further evidence that PAI‐1 plays a role in the endothelial–podocyte cross‐talk, we performed *in vitro* experiments using human primary glomerular endothelial cells (GEnC) that were cultured through iterative passages in order to induce replicative senescence. Senescence was confirmed by the observation that population doubling time increased from passage 8 to passage 17 (Fig 6A). Furthermore, only cells from late passages displayed SA‐βGal activity (Fig 6B). Similarly, *p16* mRNA expression levels increased from passage 9 (p9) to passage 17 (p17; Fig 6C). Consistently, the expression of PAI‐1, IL‐6, and IL‐8, three SASP molecules, was higher in cells from late passages as compared to cells from early passages (Fig 6C). Supernatants from these cells were collected at each passage and used to stimulate immortalized human podocytes (Fig 6D). Remarkably, we observed that only the supernatants from senescent GEnC induced morphological alterations in differentiated podocytes. In particular, the F‐actin cytoskeleton was completely reorganized, and the number of focal adhesion processes decreased as compared to control cells or cells incubated with early passage supernatants (Fig 6E). More importantly, we showed that PAI‐1 was mandatory in triggering these changes, as preincubation of senescent supernatants with tiplaxtinin, a PAI‐1 inhibitor, completely reversed the pathological phenotype. In fact, the number of focal adhesions was similar to that of controls (Fig 6E). To further prove that PAI‐1 is sufficient to injure podocytes, we treated human podocytes with recombinant human PAI‐1. Results confirmed that PAI‐1 stimulation led to the detachment of differentiated podocytes (Fig EV4). Finally, to investigate whether senescent endothelial cells trigger podocyte apoptosis, we performed FACS experiments using Annexin‐V on podocytes incubated with supernatants from endothelial cells at early (p9) or late (p17) passage, in the presence, or not, of tiplaxtinin. Interestingly, we observed an increase in apoptosis of podocytes incubated with late passage (senescent) supernatants as compared to early passage supernatants. More importantly, the increase was partially prevented by incubation with tiplaxtinin (Fig 6F). Altogether our *in vivo* and *in vitro* data indicate that senescent endothelial cells determine the fate of podocytes during aging, *via* PAI‐1 secretion.

### Glomerular endothelial cell senescence is associated with poor renal outcomes in humans

**Figure 7:**
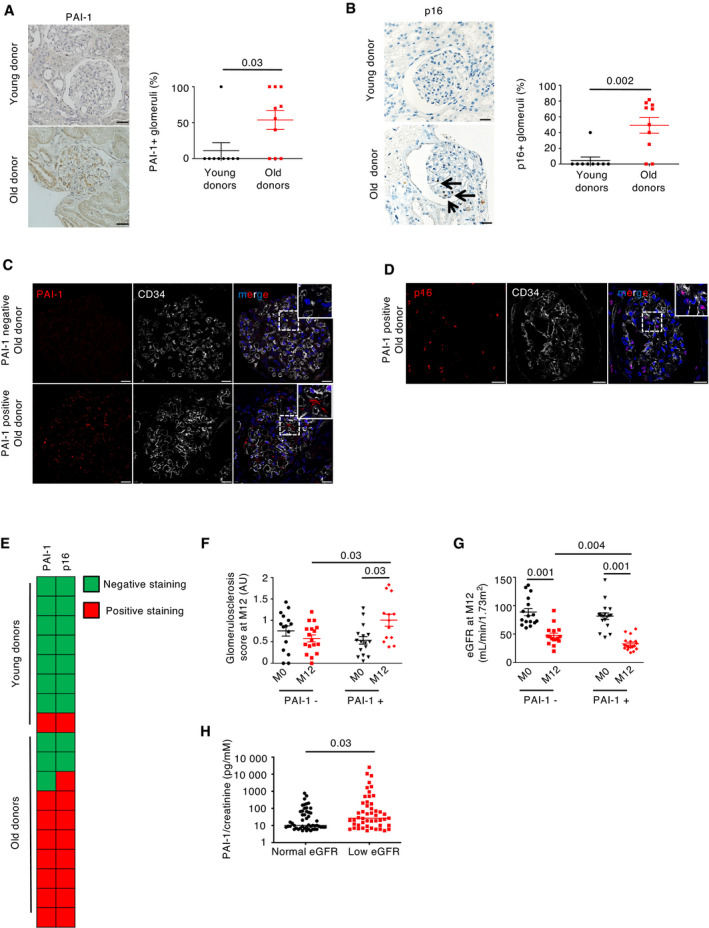
Glomerular PAI‐1 expression correlates with glomerular senescence and predicts outcomes of kidney transplant from elderly donors PAI‐1 staining (left panel) and quantification (right panel) in glomeruli of kidney biopsies from young (< 40 years of age, *n* = 8) or old (> 80 years of age, *n* = 10) kidney donors at time of transplantation (M0). Original magnification ×400. Scale bar = 20 μm.p16 staining (left panel) and quantification (right panel) in glomeruli of kidney biopsies from young (< 40 years of age, *n* = 8) or old (> 80 years of age, *n* = 10) kidney donors at time of transplantation (M0). The arrows point to the staining. Original magnification ×400. Scale bar = 20 μm.PAI‐1/CD34 coimmunostaining in old‐positive or old‐negative glomerular PAI‐1 donors. Original magnification ×630. Scale bar = 10 μm.p16/CD34 coimmunostaining in old‐positive glomerular PAI‐1 donors. Original magnification ×630. Scale bar = 10 μm.Representation of PAI‐1 and p16 staining results at time of transplantation in young (*n* = 8) or old donors (*n* = 10). Each line represents one patient. Green color is used for negative staining, and red color, for positive staining.Quantification of the glomerulosclerosis score at time of (M0), or 12 months after (M12), kidney transplantation from elderly donors in patients displaying a negative (PAI‐1^−^, *n* = 17) or positive (PAI‐1^+^, *n* = 13) glomerular PAI‐1 staining at time of transplantation (M0).Estimated glomerular filtration rate (eGFR) at time of (M0), or 12 months after (M12), kidney transplantation in patients displaying a negative (PAI‐1^−^, *n* = 17) or positive (PAI‐1^+^, *n* = 18) glomerular PAI‐1 staining at time of transplantation (M0).Urinary PAI‐1 measured by ELISA in a cohort of elderly patients with (*n* = 48) or without (*n* = 48) chronic kidney disease. Data information: Data are means ± SEM. Statistical analysis: Student's *t*‐test for (A), (B), and (H), ANOVA followed by the Tukey–Kramer test for (F) and (G).

In order to extend our experimental findings to humans, we first investigated whether the expression of PAI‐1 changes with age. To this end, we examined a first cohort of kidney transplant patients, as kidney allograft biopsies are systematically performed immediately before transplantation in our clinical department. We compared PAI‐1 expression in kidneys from young (< 40 years of age) and old (> 80 years of age) donors at the time of transplantation. Interestingly, at this time point, renal function was normal in all donors, regardless of age, and none showed pathological proteinuria or diabetes (Table 1). Moreover, cold ischemia time was comparable (Table 1). Results showed that PAI‐1 was expressed in 55% of glomeruli in kidneys from old donors as compared to 11% of glomeruli from young donors (Fig 7A). Similarly, in kidneys from old donors, 49% of glomeruli were p16‐positive, whereas only 4% of glomeruli were positive in young donors (Fig 7B). Costaining experiments between PAI‐1 and CD34 showed that PAI‐1 is secreted in close vicinity to glomerular endothelial cells in biopsies from old donors (Fig 7C). Consistently, in the same biopsies, p16 colocalized with CD34 (Fig 7D). Moreover, we observed that PAI‐1 staining matched with p16 staining (Fig 7E), supporting the idea that PAI‐1 is secreted by senescent cells.

**Table 1:** Comparison between young and old donors at time of transplantation.

	Young donors (*n* = 8)	Old donors (*n* = 10)	*P*
Donor age, years	30 (26–35)	86 (84–86)	< 0.001
Males, *n* (%)	7 (78%)	9 (90%)	1.00
Donor plasma creatinine, μmol/l	54 (49–69)	64 (60–77)	0.33
Donor proteinuria, g/l	0.10 (0.05–0.53)	0.17 (0.10–0.24)	0.25
Cold ischemia time, min	1,037 (874–1,196)	923 (788–1,310)	0.85

Data are expressed as median with interquartile (IQ25–IQ75) except if indicated. Fisher's exact test and Mann–Whitney *U*‐test were used for the comparison of qualitative and continuous variables, respectively.

IQR, interquartile range.

We next wondered whether PAI‐1 glomerular expression may predict allograft outcomes. Toward this goal, we studied a second cohort of 35 kidney transplant recipients who received a kidney from an old donor (> 80 years of age; Table 2). Each recipient had a protocol kidney biopsy at the time of transplantation (M0), as well as 12 (M12) months after transplantation. Of note, since our aim was to determine whether PAI‐1 staining could be considered as an early predictive biomarker of graft outcome, we selected patients with normal renal morphology at time of transplantation. Among the 35 donors, 18 (51%) showed glomerular PAI‐1 staining at time of transplantation. Remarkably, glomerular PAI‐1 expression at the time of transplantation predicted the outcome of kidney allografts. In fact, recipients with PAI‐1‐positive glomeruli developed more severe glomerulosclerosis 12 months after transplantation (Fig 7F and Table 3). Similarly, allograft function, as judged by eGFR, was significantly lower in PAI‐1‐positive patients as compared to PAI‐1‐negative patients 12 months after transplantation (Fig 7G and Table 3). A careful examination of clinical characteristics known to affect graft outcomes, such as cold ischemia, pre‐allograft sensitization, acute rejections, donor kidney function, interstitial fibrosis, and glomerulosclerosis at time of transplantation, revealed that none of these differed between PAI‐1‐positive and PAI‐1‐negative patients (Table 2 and Table 3). In addition, a multivariate analysis revealed that glomerular PAI‐1 staining at the time of transplantation was independently associated with eGFR at M12 as compared to other factors known to affect graft outcome (Appendix Table S1). Hence, PAI‐1 seems to be a promising prognostic biomarker of kidney transplant from elderly donors.

**Table 2:** Clinical data in PAI‐1‐positive and PAI‐1‐negative kidney recipients at time of transplantation.

	PAI‐1‐negative glomeruli (*n* = 17)	PAI‐1‐positive glomeruli (*n* = 18)	*P*
Recipient age, years	70 (65–73)	69 (67–72)	0.92
Donor age, years	82 (81–84)	82 (80–83)	0.36
Males, *n* (%)	7 (41%)	11 (61%)	0.32
HLA donor‐specific antibody, *n*	0	0	1.00
Cold ischemia time, min	1,101 (964–1,440)	1,120 (810–1,620)	0.65
Donor eGFR (ml/min/1.73 m^2^)	82 (71–120)	82 (72–87)	0.66
Delayed graft function, *n* (%)	3 (18%)	3 (17%)	1.00
Dual transplantation, *n* (%)	11 (65%)	14 (78%)	0.53
Immunosuppressive treatment			
Tacrolimus, *n* (%)	13 (77%)	13 (72%)	1.00
Cyclosporine, *n* (%)	4 (24%)	5 (28%)	1.00
Steroids, *n* (%)	17 (100%)	18 (100%)	1.00
Mycophenolic acid, *n* (%)	17 (100%)	18 (100%)	1.00
Morphological data at M0			
Glomerulosclerosis score, AU	0.9 (0.3–1)	0.5 (0.2–0.7)	0.14
Severe arteriosclerosis, *n* (%)	7 (41%)	12 (66.7%)	0.18
Severe interstitial fibrosis, *n* (%)	7 (41%)	12 (66.7%)	0.29

Negative and positive glomerular PAI‐1 staining was performed on donor kidney biopsies at time of transplantation. AU, arbitrary unit; eGFR, estimated glomerular filtration rate; M0, time of transplantation; Tx, transplantation. Severe arteriosclerosis and severe interstitial fibrosis were defined as CV3 and IF/TA3 according to the Banff classification. Data are expressed as median with interquartile (IQ25–IQ75) except if indicated. Fisher's exact test and Mann–Whitney *U*‐test were used for the comparison of qualitative and continuous variables, respectively.

**Table 3:** Clinical data in PAI‐1‐positive and PAI‐1‐negative kidney recipients 12 months after transplantation.

	PAI‐1‐negative glomeruli (*n* = 17)	PAI‐1‐positive glomeruli (*n* = 18)	*P*
Post‐Tx diabetes, *n* (%)	5 (29%)	6 (33%)	1.00
BMI, kg/m^2^	24 (20–25)	24 (22–26)	0.40
Acute rejection, *n* (%)	3 (18%)	3 (17%)	1.00
BK viremia, *n* (%)	1 (6%)	1 (6%)	1.00
Plasma creatinine, μmol/l	109 (90–130)	155 (129–182)	< 0.001
eGFR, ml/min/1.73 m^2^	45.6 (40–56)	29.1 (25–37)	< 0.001
Morphological data			
Glomerulosclerosis score, AU	0.55 (0.31–0.82)	1.00 (0.55–1.43)	< 0.05
Severe arteriosclerosis, *n* (%)	11 (65%)	9 (50%)	0.50
Severe interstitial fibrosis, *n* (%)	10 (59%)	8 (44%)	0.51

PAI‐1 staining to define positive and negative glomeruli was performed on donor kidney biopsies at time of transplantation. AU, arbitrary unit; BMI, body mass index; eGFR, estimated glomerular filtration rate; Tx, transplantation. Severe arteriosclerosis and severe interstitial fibrosis were defined as CV3 and IF/TA3 according to the Banff classification. Data are expressed as median with interquartile (IQ25–IQ75) except if indicated. Fisher's exact test and Mann–Whitney *U*‐test were used for the comparison of qualitative and continuous variables, respectively.

### Urinary PAI‐1 is associated with aging nephropathy in humans

We finally wondered whether urinary PAI‐1 excretion correlates with age‐related CKD in native kidneys. To this end, we established a third cohort of patients (non‐transplanted) consecutively admitted in a geriatric unit from a tertiary healthcare facility and collected urine samples to measure PAI‐1 level by ELISA. We excluded patients with diabetes mellitus, since diabetes has been shown to increase urinary PAI‐1 excretion (Verhave *et al*, 2013). We collected urine from 96 patients, among which 48 (50%) displayed reduced renal function. Patients with or without CKD were comparable for factors that might potentially modify PAI‐1 expression, such as active cancer, metabolic syndrome, obesity, anticoagulant therapy, or renin–angiotensin system blockers (Table 4). Patients with normal renal function (defined as an eGFR > 60 ml/min/1.73 m^2^) have a median eGFR of 76 ml/min/1.73 m^2^ (IQR: 68–88), whereas patients with reduced renal function (defined as an eGFR < 60 ml/min/1.73 m^2^) have a median eGFR of 41 ml/min/1.73 m^2^ (IQR: 34–50). Interestingly, we observed that patients with reduced renal function had statistically significantly higher urinary PAI‐1 excretion levels as compared to patients with normal renal function (Fig 7H and Table 4). Together, these data suggest that PAI‐1 might be involved in human aging nephropathy.

**Table 4:** Clinical and biological data of the “aging cohort”.

	Normal eGFR (*n* = 48)	Low eGFR (*n* = 48)	*P*
Age, years	90 (85–92)	90 (86–95)	0.13
Males, *n* (%)	19 (40%)	21 (44%)	0.83
BMI, kg/m^2^	22.4 (19.9–25.6)	22.5 (20.2–24.0)	0.25
HBP, *n* (%)	31 (65%)	36 (75%)	1.00
Metabolic syndrome, *n* (%)	6 (13%)	7 (15%)	0.27
Anticoagulant therapy, *n* (%)	13 (27%)	17 (35%)	0.63
History of neoplasia, *n* (%)	20 (42%)	25 (52%)	0.36
RAS blockers, *n* (%)	20 (42%)	22 (46%)	0.65
Serum creatinine, μmol/l	67 (58–77)	117 (100–165)	< 0.001
eGFR, ml/min/1.73 m^2^	76 (68–88)	41 (34–50)	< 0.001
Urinary Pu/Creat, mg/mmol	24 (14–52)	33 (15–122)	0.30
Urinary PAI‐1/Creat, pg/mmol	10 (8–65)	27 (10–196)	< 0.05

Data are expressed as median with interquartile (IQ25–IQ75) except if indicated. Fisher's exact test and Mann–Whitney *U*‐test were used for the comparison of qualitative and continuous variables, respectively.

BMI, body mass index; Creat, creatinine; eGFR, estimated glomerular filtration rate; HBP, high blood pressure; IQR, interquartile range; PAI‐1, plasminogen activator inhibitor 1; Pu, proteinuria; RAS, renin–angiotensin system.

## Discussion

It is well established that with age, kidneys develop lesions, in particular glomerulosclerosis. However, the molecular mechanisms involved in the deterioration process are unclear. It has been suggested that senescence could play a role, but the pathophysiological link between senescence and glomerular lesion development remains unexplained. By combining *in vivo* experimental models of aging with genetically modified mice and *in vitro* studies, we discovered a detrimental cross‐talk between senescent endothelial cells and podocytes. Among the possible mediators of this cross‐talk, PAI‐1 seems to play a key role. *In vivo*, deletion of *PAI‐1* in endothelial cells blunted the development of glomerulosclerosis in aged mice by decreasing podocyte loss. *In vitro*, preincubation of supernatants from senescent endothelial cells with tiplaxtinin, a PAI‐1 inhibitor, prevented podocyte loss. Consistently, depletion of senescent cells prevented podocyte loss in old *p16 INK‐ATTAC* transgenic mice. More importantly, we provided evidence that these data are clinically relevant. In fact, glomerular PAI‐1 staining was predictive of kidney allograft dysfunction 12 months after transplantation from elderly donors. Moreover, we observed that PAI‐1 excretion was increased in the urine of elderly patients with recognized aging nephropathy as compared to age‐matched patients without renal impairment. In conclusion, our study reveals the critical role played by endothelial senescence in the development of glomerular lesions during aging, and identified PAI‐1 as a novel promising biomarker for the prediction of kidney dysfunction in patients receiving a kidney from elderly donors.

Several studies have shown that the number of senescent cells increases in the kidney with age and that this increase is associated with the development of renal lesions (Baker *et al*, 2016; Valentijn *et al*, 2018). Moreover, it has been observed that systemic depletion of senescent cells prevented the development of the age‐related glomerulosclerosis (Baker *et al*, 2016). Intriguingly, while senescence affects primarily tubular cells, lesions mostly develop in glomeruli with age (Melk *et al*, 2004; Baker *et al*, 2016). However, in pathological contexts characterized by glomerular lesions, i.e., diabetic nephropathy, focal segmental glomerular sclerosis, or glomerulonephritis, it has been shown that senescence may involve podocytes, and mesangial, endothelial, or parietal epithelial cells (Valentijn *et al*, 2018). Recently, it has been demonstrated that senescence may affect endothelial cells in the liver and kidney under physiological and pathological conditions (Omori *et al*, 2020). Differences in the cellular mechanisms underlying these pathological contexts may explain this discrepancy. Alternatively, it is possible that technical issues explain these differences, i.e., the difficulty in identifying senescent glomerular cells. Our results are in favor of this hypothesis. Using different complementary markers, we clearly showed that glomerular endothelial cells become senescent with age, suggesting that these cells might play a role in the development of glomerular lesions. Consistent with this idea, we demonstrated that the selective inhibition of PAI‐1 in endothelial cells was sufficient to protect the kidney from lesion development in aged mice. Moreover, we observed that depletion of senescent cells in *p16 INK‐ATTAC* mice resulted in the decrease in both PAI‐1 expression and podocyte loss. It is thus tempting to propose that activation of senescence in glomerular endothelial cells is the first step of a cascade of events leading to glomerulosclerosis in aging nephropathy.

Podocyte loss has been involved in the development of glomerulosclerosis during aging (Steffes *et al*, 2001; Appel *et al*, 2009). Our data confirmed that increased glomerular apoptosis and podocyte loss accompanied the development of glomerular lesions in aged mice. However, since senescence seems to affect only endothelial cells, we hypothesized that a pathological cross‐talk between these cells and podocytes underlies the development of glomerular lesions. Communication between these two cell types has already been reported. It has been shown that podocytes signal to glomerular endothelial cells in a paracrine manner via VEGF, angiopoietins, endothelins, or chemokines (Dimke *et al*, 2015; Bartlett *et al*, 2016). On the contrary, it has been reported that endothelial cells may communicate with mesangial cells *via* PDGFβ secretion (Bjarnegård *et al*, 2004; Eng *et al*, 2005). To the best of our knowledge, our study is the first one reporting a communication from glomerular endothelial cells to podocytes. Whether this cross‐talk is involved in other pathological contexts is an interesting idea that deserves further studies.

We identified PAI‐1 as a mediator of the detrimental cross‐talk between endothelial cells and podocytes. Although it has been reported that PAI‐1 leads to podocyte damage in several experimental models of CKD (Rondeau *et al*, 1990; Yamamoto *et al*, 1993; Grandaliano *et al*, 2000; Lee *et al*, 2001; Hamano *et al*, 2002; Paueksakon *et al*, 2002), as well as in humans (Eddy, 2002), the molecular mechanisms by which PAI‐1 induces podocyte loss are not yet clear. A recent study suggested that PAI‐1 might act on podocytes through its interaction with the urokinase receptor (uPAR) via the uPA complex. Once bound to uPAR, the PAI‐1/uPA complex leads to β1‐integrin endocytosis that in turn triggers podocyte detachment (Kobayashi *et al*, 2015). The observation that tiplaxtinin, a PAI‐1 inhibitor (Elokdah *et al*, 2004), prevented the loss of focal adhesions (a marker of cell detachment) and apoptosis in podocytes incubated with supernatants from senescent endothelial cells strongly supports this idea. However, we cannot exclude the possibility that PAI‐1 acts through, or in combination with, other mechanisms. In particular, PAI‐1 could act by modulating other SASP components, since we observed that *PAI‐1* inactivation resulted in decreased glomerular senescence in aged *PAI‐1^Δendo^* mice. Along the same line, we cannot exclude that by modifying metalloprotease activities, PAI‐1 participates in the development of glomerulosclerosis (Ramos‐DeSimone *et al*, 1999; Oh *et al*, 2002).

Organ shortage for kidney transplantation is a major public health problem. Nearly 5,000 people in the United States and more than 3,000 people in Europe die each year while waiting for a kidney transplant (Hart *et al*, 2017; ). Over the past decade, the worldwide paucity of available donated kidneys has pushed to expand the criteria for organ supply. In particular, this has led to the consideration of organs from older donors. Recent studies suggest that the outcomes of kidneys from old donors are similar to those of kidneys from young donors when clinical and histological criteria are respected (Aubert *et al*, 2015; Ruggenenti *et al*, 2017). In particular, it has been shown that these grafts are suitable for elderly people, who constitute one third of patients currently awaiting kidney transplantation (Aubert *et al*, 2015). Thus, it appears that the success of kidney transplantation is more related to the criteria of the organ than to the age of the donor. In this regard, our data bring an important contribution. In fact, we have identified a sensitive method, i.e., PAI‐1 immunostaining on preimplantation biopsies, to predict whether kidneys from elderly donors are at an increased risk of functional decline. It is worth noting that preimplantation biopsies displayed a normal morphology in all patients, supporting the idea that PAI‐1 is an early predictive biomarker.

By studying a cohort of elderly people with native kidneys, we demonstrated that increased urinary PAI‐1 excretion is associated with age‐related CKD. Interestingly, a rare loss‐of‐function mutation in *SERPINE1*, the gene encoding PAI‐1, has been associated with longevity (Khan *et al*, 2017). It is well known that that the consequences of aging, i.e., aging nephropathy, vary among individuals (Eriksen & Ingebretsen, 2006; Wetzels *et al*, 2007). Several genetic, epigenetic, and environmental factors account for this heterogeneity (Rowland *et al*, 2019). Our data suggest that, among these, PAI‐1 may play a role by predisposing to renal deterioration. Hence, levels of PAI‐1 may identify, among aged individuals, those that will develop renal failure. It is worth noting that other SASP molecules, such as IL‐6, IL‐18, or MMP9, have been shown to be excreted in urines of CKD patients (Wolkow *et al*, 2008; Musiał *et al*, 2015; Lipiec *et al*, 2018; Bullen *et al*, 2021). Whether they participate with PAI‐1 in the deterioration process is an interesting hypothesis that deserves to be tested in further studies.

In conclusion, this work has identified PAI‐1 as a critical mediator of an endothelial–podocyte cross‐talk that leads to glomerular lesions during aging in both mice and humans. In this context, the activation of a senescence program in endothelial cells is necessary. Disruption of this pathological pathway prevents the renal deterioration process in aged mice. Moreover, PAI‐1 is able to predict CKD progression in transplanted patients with kidneys from elderly donors. Therefore, PAI‐1 may be both a prognostic marker and a therapeutic target to prolong renal survival during aging.

## Materials and Methods

### Animals

Mice used for experiments were male and female C57BL/6, *PAI‐1^flox^* ^/^ *^flox^*, *VeCadh‐Cre* ^+/−^, *p16 INK‐ATTAC*, and *p16* ^+/^ *^luc^* (Alva *et al*, 2006; Rannou *et al*, 2015; Baker *et al*, 2016; Soysouvanh *et al*, 2020). C57Bl/6 mice were purchased from Janvier Laboratories. *PAI‐1^flox^* ^/^ *^flox^* mice were generated and breed in Dr Milliat's laboratory. *VeCadh‐Cre* ^+/−^ were purchased from Jackson laboratories (ref B6.FVB‐Tg(Cdh5‐cre)7Mlia/J). *p16* ^+/^ *^luc^* mice were a gift from Dr Adnot (Institut Mondor de Recherche Biomédicale, Creteil, France). p16 INK‐ATTAC mice were generated and breed in Dr Baker's laboratory. *PAI‐1^flox^* ^/^ *^flox^* mice were crossed with *VeCadh‐Cre* ^+/−^ to generate *PAI‐1^Δendo^* mice, in which *PAI‐1* (*Serpine1* gene) was selectively deleted in endothelial cells. Mice were fed *ad libitum* and housed at constant ambient temperature in a 12‐h light, 12‐h dark cycle. Animal procedures were approved by the “Services Vétérinaires de la Préfecture de Police de Paris”, by the “Ministère de l'Enseignement Supérieur de la Recherche et de l'Innovation” and by the ethical committee of the Paris Descartes University.

### Experimental protocol

#### Physiological aging protocol

Control mice were sacrificed at 4 (young mice, *n* = 6), 12 (*n* = 4), 18 (*n* = 4), and 24 (aged mice, *n* = 6) months of age. *PAI‐1^Δendo^* mice (*n* = 12) and their wild‐type controls (*PAI‐1^flox^*, *n* = 12) were sacrificed at 22 months of age. Kidneys were collected for morphological analyses, and protein and mRNA studies.

#### Irradiation protocol

Control mice underwent (*n* = 5) or not (*n* = 4) sublethal total body irradiation of eight grays at 6 weeks of age and sacrificed 12 months later. Kidneys were collected as described above.

#### Depletion of p16‐positive cell protocol

*p16 INK‐ATTAC* male mice were treated from 12 to 28 months with either AP20187 (2 mg/kg biweekly by intraperitoneal injection; *n* = 5) or vehicle (*n* = 5). Mice were sacrificed at 28 months, and kidneys were collected as described above.

#### p16‐luciferase mouse protocol

*p16* ^+/^ *^luc^* mice experienced the physiological aging (*n* = 3) or irradiation (*n* = 3–6) protocol as described above. Transgene activation, an index of p16 expression, was visualized by bioluminescence. Briefly, 4.5 mg of luciferin (d‐Luciferin, K^+^ Salt, Interchim) was injected intraperitoneally before the acquisition of emitted bioluminescence (BLI; ph/s/cm²/sr) by a CCD (charge‐coupled device) camera (PhotonIMAGER Optima—Biospace). Bioluminescence was measured at 6, 12, 18, and 24 months of age or once a month from 3 to 13 months after irradiation.

### Clinical samples

Two studies were conducted on kidney transplant recipients followed at the Renal Transplant Department of Necker Hospital. For the first study, eight patients receiving a kidney from young donors (< 40 years of age) and 10 patients receiving a kidney from older donors (> 80 years of age) were compared. All patients underwent a protocol kidney allograft biopsy immediately before transplantation (M0). Patient demographic and clinical characteristics are listed in Table 1. For the second study, 35 patients who received a kidney from an older donor (> 80 years of age) were studied. All patients underwent a protocol kidney biopsy at the time of transplantation (M0) and 3 and 12 months after transplantation. At these time points, urine and blood were also collected. Based on glomerular PAI‐1 staining at the time of transplantation, patients were divided into two groups: (i) patients with positive glomerular PAI‐1 staining (*n* = 18) and (ii) patients with negative glomerular PAI‐1 staining (*n* = 17). The two groups were compared. Patient demographic and clinical characteristics are listed in Tables 2 and 3. The protocol was approved by the Institutional Review Board of Necker Hospital; informed written consent was obtained from each patient.

For the aging kidney cohort, we consecutively collected urine (under protease inhibitors) from patients with (*n* = 48) or without (*n* = 48) suspected age‐related CKD, admitted in the geriatric unit of Hôpital Européen Georges Pompidou, Paris, France. We excluded patients with diabetes mellitus and overt malignancies, as these conditions have been shown to increase PAI‐1 expression. First morning, urine was collected and stored at 4°C, before being centrifuged at 1,000 *g* for 15 min. Clinical–demographic data of these patients are recapitulated in Table 4.

The experiment conformed to the principles set out in the WMA Declaration of Helsinki and the Department of Health and Human Services Belmont Report.

### Cell cultures

Human primary glomerular endothelial cells (GEnC) were purchased from Cell System and cultured according to the manufacturer's instructions. For each passage, supernatant was collected and stored at −80°C after centrifugation at 500 *g* for 5 min. Population doubling time was assessed by counting the number of cells in a six‐well plate each day. For SA‐βGal staining, GEnC were fixed in 4% PFA before performing the staining as indicated below.

Human immortalized podocytes, a gift from Dr Saleem (University of Bristol, UK), were cultured as previously described (Saleem *et al*, 2002). Briefly, cells were maintained in RPMI 1640 medium supplemented with 10% FBS and 100 μg/ml penicillin/streptomycin. To propagate podocytes, cells were grown at 33°C. To induce differentiation, podocytes were grown at 37°C for 14 days. All reagents were purchased from Thermo Fisher Scientific. All cell cultures were mycoplasma‐negative.

Differentiated podocytes were incubated with recombinant human PAI‐1 at 5 nM (Bio‐Techne) for 30 min or with supernatants from GEnC at passage 7 or 9 (young cells) or passage 17 (senescent cells) for 72 h. PAI‐1 inhibition was achieved by preincubating GEnC supernatants with 5 mM tiplaxtinin (Sigma‐Aldrich, PZ0295) at 37°C for 1 h before incubation with podocytes for 72 h. Podocytes were then used for immunofluorescence, detachment, and apoptosis studies, as described below.

### Urine and plasma analyses

For patients, urinary protein and creatinine concentrations were determined using an Architect C16000 multiparametric analyzer (Abbott), whereas plasma creatinine was evaluated using an AU5800 (Beckman Coulter) autoanalyzer. For both analyzers, creatinine determination was performed using the enzymatic IDMS method. The estimated glomerular filtration rate was calculated using the CKD‐EPI equation (Levey *et al*, 2009). For ELISA analyses, urine samples were first concentrated 40–50× using Ultra‐2 30k centrifugal filter devices (Millipore UFC203024). ELISA for human PAI‐1 (R&D Systems DSE100) was performed according to the manufacturer's instructions. The absorbance was measured using a Multiskan Sky (Thermo Scientific) microplate reader.

For mouse samples, serum creatinine concentrations were measured using an Olympus multiparametric analyzer (Instrumentation Laboratory).

### Histological analysis

Mouse kidneys from all protocols except INK‐ATTAC were fixed in 4% paraformaldehyde and paraffin‐embedded, and 4‐μm sections were stained with periodic acid–Schiff (PAS). *p16 INK‐ATTAC* mouse kidneys were fixed in 10% neutral buffered formalin. Images were acquired using a Nikon Digital Camera Dx/m/1200. All sections were evaluated by a renal pathologist who was unaware of the group studied. The degree of glomerular lesions was evaluated at a magnification of ×400 from 10 microscopic fields using the following scoring system: 0 = no lesion, 1 = mild sclerosis, affecting up to 25% of the glomerulus, 2 = moderate sclerosis, affecting 25–50% of the glomerulus, and 3 = severe sclerosis affecting 50–75% of the glomerulus.

Human transplant biopsies were fixed in an alcohol–formalin–acetic acid solution (AFA) and embedded in paraffin. Sections (4 μm) were stained with PAS, Masson's trichrome, and hematoxylin and eosin. All sections were evaluated by a renal pathologist who was unaware of the group studied. Each biopsy was examined according to the Banff '15 scoring system (Loupy *et al*, 2017). Glomerulosclerosis score was quantified as described above for mice.

### Immunohistochemistry and immunofluorescence

For mouse samples, 4‐μm sections of paraffin‐embedded kidneys underwent antigen retrieval by high‐pressure heating in a solution of Tris base, EDTA, and Tween (p21, 53BP1, CD34, WT1) or in a citrate solution (PAI‐1) for 15 min. Sections were incubated with the following primary antibodies: mouse monoclonal anti‐p21 (BD Bioscience, ref 556431) at 1/200, rat monoclonal anti‐CD34 (eBioscience, ref 14‐0341‐85) at 1/50 (mouse sections), mouse monoclonal anti‐CD34 (Dako, ref M7165) at 1/100 (human sections), griffonia simplicifolia‐FITC (Vector, ref FL‐1101) at 1/50, monoclonal guinea‐pig anti‐nephrin (Progen, ref GP‐N2) at 1/100, monoclonal rabbit anti‐pH2AX (Cell Signaling, ref 9718S) at 1/200, rabbit polyclonal anti‐53BP1 (Abcam, ref ab36823) at 1/100, mouse monoclonal anti‐WT1 (Wilms Tumor 1) (Dako, ref M3561) at 1/50, and rabbit polyclonal anti‐PAI‐1 (Abcam, ref ab28207) at 1/100. Sections were incubated with the primary antibody overnight at 4°C. After washing, sections were then incubated with the appropriate secondary antibody: biotin‐coupled anti‐rat (Vector Laboratories) at 1/200 for CD34 (mice sections), anti‐rabbit Alexa Fluor 488 (Thermo Fisher Scientific) at 1/400 for 53BP1, PAI‐1, or pH2AX, anti‐mouse Alexa Fluor 555 at 1/200 for CD34 (human section, Thermo Fisher Scientific), anti‐guinea‐pig Alexa Fluor 555 at 1/200 for nephrin (Thermo Fisher Scientific), anti‐mouse coupled with biotin (Vector Laboratories) at 1/200 and at 1/500 for p21 and WT1, respectively, and anti‐rabbit coupled to HRP (GE Healthcare) at 1/300 for PAI‐1.

DAB staining was used to detect HRP‐coupled secondary antibodies. For immunohistochemistry, sections were counterstained with hematoxylin, while immunofluorescence sections were counterstained with Hoechst.

For colocalization experiments, coimmunostaining of p21/griffonia simplicifolia/nephrin, pH2AX/griffonia simplicifolia, 53BP1/CD34, p21/CD24, and PAI‐1/griffonia simplicifolia in mouse sections, and PAI‐1/CD34 and p16/CD34 in human sections was performed as indicated above, except that the different primary or secondary antibodies were pooled together. The number of endothelial senescent cells was quantified by counting double‐positive cells p21/griffonia simplicifolia, pH2AX/griffonia simplicifolia, 53BP1/CD34, and p21/CD34 at ×600 magnification on 10 randomly selected glomeruli.

The number of podocytes was determined by counting the number of WT1‐positive cells per glomerulus in 10 microscopic fields at a magnification of ×600.

p21 staining was quantified as the number of p21‐positive endothelial cells per glomerulus in 10 microscopic fields at a magnification of ×600.

The number of 53BP1 and pH2AX spots was counted in glomerular endothelial cells and adjusted to number of glomerular nuclei using ImageJ.

PAI‐1 labeling was quantified as the percentage of positive glomeruli over the total number of glomeruli in 10 microscopic fields at ×600 magnification.

For human samples, p16 staining was performed with the automat BenchMark ULTRA instrument (Ventana) using CINtec^®^ Histology Staining Kit and OptiView DAB IHC Detection Kit. PAI‐1 staining was performed using the same protocol as in mice. p16 labeling and PAI‐1 labeling were quantified as the percentage of positive glomerular staining on the total number of glomeruli in the section. Differentiated podocytes were fixed in 4% paraformaldehyde for 15 min, permeabilized in PBS containing Triton X‐100 0.1% and BSA 3%, and then incubated with anti‐paxillin antibody (BD Bioscience, ref 610051) at 1/2,000 and phalloidin‐TRITC (Sigma, ref P1951) at 1/1,000.

Immunofluorescence studies were analyzed using the Zeiss LSM 700 confocal microscope. Quantification of focal adhesions, characterized by paxillin‐ and phalloidin‐positive staining, was performed using ImageJ software. Briefly, 30 independent cells from three independent experiments were used for quantification. After using a band‐pass filter, focal adhesions were automatically counted by using the “analyze particle” tool of ImageJ software.

### *In vivo* p16 activity by bioluminescence

After intraperitoneal injection of luciferin in *p16* ^+/^ *^luc^* mice, bioluminescence (BLI) was acquired using a CCD camera. For both protocols, BLI of the kidney area was measured. For the aging protocol, we expressed the results as the ratio of the kidney area BLI over body weight, since the protocol lasted for almost 2 years, implying a change of mice morphology. For the irradiation protocol, since the mice received total body irradiation, we expressed the results as the kidney area BLI over the total body area BLI, limiting the background effect. BLI was measured using the Photovision software (Biospace Lab).

### Apoptosis assay

*In vivo,* apoptosis was detected in 4‐μm sections of paraffin‐embedded kidneys by TUNEL assay using the *In situ* Cell Death Detection Fluorescein Kit (Roche ref 11684795910) according to the manufacturer's protocol. The glomerular apoptotic index was calculated as the number of glomeruli with at least one TUNEL‐positive nucleus over the total number of glomeruli. All the microscopic fields of a whole kidney section were quantified.

*In vitro,* apoptosis of podocytes was evaluated using Annexin‐V staining according to the manufacturer's instruction. Briefly, after a 72‐h incubation with supernatants from young or senescent GeNC in the presence, or not, of tiplaxtinin, cells were detached, counted, and stained with Annexin‐V‐FITC. Quantification of Annexin‐V‐FITC‐positive cells was performed by FACS, using a Sony SP6800.

### Beta galactosidase staining (SA‐βGal)

Five‐micrometer frozen kidney sections or cells on coverslips were fixed in a solution of 1% paraformaldehyde. Sections were incubated at 37°C overnight with SA‐βGal labeling solution (for 10 ml of solution: 2 ml of 5X citric acid at pH 6, 1 ml of 50 mM potassium ferricyanide, 1 ml of 50 mM potassium ferrocyanide, 0.33 ml of 5 M sodium chloride, 20 μl of 1 M magnesium chloride, 0.5 ml of X‐gal diluted to 20 mg/ml in *N*,*N*‐dimethylformamide, and 5.2 ml of water). The slides were counterstained with eosin and then mounted with Fluoromount (Sigma). SA‐βGal staining was quantified by counting the number of positive glomeruli in a whole kidney section or the number of SA‐βGal‐positive cells per coverslip at a magnification of ×600.

For GeNC, cells grown on coverslips were fixed in a solution of 3% paraformaldehyde for 3 min and treated as described above.

### Podocyte detachment assays

After incubation of podocytes with recombinant PAI‐1 for 30 min, supernatants were collected. After a 5‐min centrifugation at 500 rpm, cells were suspended in 100 μl of PBS and counted using a Malassez cell. In parallel, adherent cells were trypsinized and counted using the same protocol. The ratio of cells in the supernatant to the number of adherent cells was considered as the percentage of podocyte detachment.

### Quantitative real‐time polymerase chain reaction (qRT–PCR)

RNA extraction from frozen kidneys or GeNC was performed using a Macherey‐Nagel Kit according to the manufacturer's recommendations. Reverse transcription was performed on 1 μg of RNA. Quantitative real‐time PCR was performed using the SYBR Green method and CFX96 Touch™ Real‐Time PCR Detection System (Bio‐Rad). Primer sequences (Eurogentec) are provided in Appendix Table S2.

### Statistical analysis

Data are expressed as the mean ± SEM (standard error of the mean). For human samples, data are expressed as the median with interquartile (IQ25–IQ75). Differences between the experimental groups were evaluated using analysis of variance (ANOVA) followed by the Tukey–Kramer test. When only two groups were compared, the Mann–Whitney test was used. The results are representative examples of more than three independent experiments. No randomization or blinding was done. No animals were excluded from the analysis. *P* < 0.05 was considered as significant. The statistical analyses were performed using GraphPad Prism software. Multivariate analysis consisted in multiple linear regression between eGFR at M12 and suspected M0 factors associated with M12 eGFR. These factors were selected based on the literature and our data, and after bivariate analysis. Analysis was performed using R version 4.0.3 (2020‐10‐10).

## Author contributions

CC designed and performed the experiments and analyzed the data. OLG, FS, and LA also performed experiments and analyzed the data. FV and TN‐K performed the human studies. MT performed the *in vitro* studies. CN performed animal experiments. OS‐F., TM, MR, JLG, DN, CL, and DA provided clinical samples. BGC and DJB provided the *p16 INK‐ATTAC* model kidney samples. CC also contributed to writing the manuscript. GF and BK revised the manuscript. FM provided the conceptual framework and revised the manuscript. FT provided the conceptual framework and designed the study, supervised the project, and wrote the manuscript.

## Conflict of interest

B.G.C. and D.J.B. are shareholders and coinventors on patent applications licensed to or filed by Unity Biotechnology, a company developing senolytic medicines, including small molecules that selectively eliminate senescent cells. Research in the Baker laboratory has been reviewed by the Mayo Clinic Conflict of Interest Review Board and is being conducted in compliance with Mayo Clinic conflict of interest policies. All other authors declare that they have no conflict of interest.

## Supporting information



Appendix

Expanded View Figures PDF

## Data Availability

This study includes no data deposited in external repositories.
