# Supplementary material for: Glomerular endothelial cell senescence drives age‐related kidney disease through PAI‐1
Source: EMBO Mol Med. 2021 Nov 2;13(11):e14146. doi: 10.15252/emmm.202114146 (PMC8573606; doi:10.15252/emmm.202114146)
Supplement: Supplementary file 1 — Appendix [file EMMM-13-e14146-s001.pdf]

Appendix table of contents:

- Appendix Table S1: Multivariate analysis of eGFR 12 months after transplantation.

- **Appendix Table S2:** List of the primers used**.**

Appendix Table S1: Multivariate analysis of eGFR 12 months after transplantation.

Variable		N	Estimate		p
**PAI–1 glomerular staining**	neg	17		Reference	
	pos	18		−16.05 (−27.89, −4.20)	0.01
**Banff chronic vascular lesion (cv) score**	0	3		Reference	
	1	12		8.90 (−10.81, 28.62)	0.36
	2	16		9.86 (−10.07, 29.80)	0.32
	3	4		4.03 (−23.37, 31.43)	0.76
**Banff interstitial fibrosis (IFTA) score**	0	15		Reference	
	1	15		−1.66 (−13.34, 10.02)	0.77
	2	4		−6.36 (−23.91, 11.19)	0.46
	3	1		−11.70 (−49.73, 26.34)	0.53
**Donor eGFR**		35		−0.05 (−0.29, 0.19)	0.68

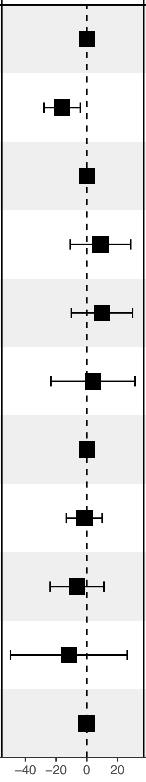

eGFR: estimated glomerular filtration rate; cv: chronic vascular lesions; IFTA: interstitial fibrosis tubular atrophy. Forest plot of estimate coefficient of multiple linear regression of eGFR at 12 months after transplantation (M12) with PAI-1 glomerular staining at time of transplantation (M0), Banff chronic vascular lesion score at M0, Banff interstitial fibrosis score at M0 and donor eGFR. $P < 0.05$ was considered as significant.

Appendix Table S2: List of the primers used.

Gene name		Sequence
Mouse GAPDH	Forward	TGCACCACCAACTGCTTAG
	Reverse	TGGATGCAGGGATGATGTT
Mouse HPRT	Forward	GGCCAGACTTTGTTGGATTTG
	Reverse	CGCTCATCTTAGGCTTTGTATTTG
Mouse RPL13	Forward	CTCATCCTGTTCCCCAGGAA
	Reverse	GGGTGGCCAGCTTAAGTTCTT
Mouse PAI-1	Forward	GTAGCACAGGCACTGCAAAA
	Reverse	CCACTGTCAAGGCTCCATCA
Mouse IL6	Forward	CTCTGGGAAATCGTGGAAATG
	Reverse	AAGTGCATCATCGTTGTTCAT
Mouse IL1b	Forward	CTGGTACATCAGCACCTCACA
	Reverse	GAGCTCCTTAACATGCCCTG
Mouse VEGFA	Forward	GCCCTGAGTCAAGAGGACAG
	Reverse	CTCCTAGGCCCCTCAGAAGT
Mouse MMP13	Forward	CTTCTTCTTGTTGAGCTGGACTC
	Reverse	CTGTGGAGGTCACTGTAGACT
Mouse p16	Forward	GGCCAATCCCAAGAGCAGAG
	Reverse	GCCACATGCTAGACACGCTA

Human p16	Forward	CAAGATCACGCAAAAACCTCTG
	Reverse	CGACCCTATACACGTTGAACTG
Human p21	Forward	TGGCTCTCAGGGTCGAAA
	Reverse	AAGATCAGCCGGCGTTTG
Human IL6	Forward	CCTGAACCTTCCAAAGATGGC
	Reverse	TTCACCAGGCAAGTCTCCTCA
Human IL8	Forward	CTTGGCAGCCTTCCTGATTT
	Reverse	GGGTGGAAAGGTTTGGAGTATG
Human PAI1	Forward	AGTGGACTTTTCAGAGGTGGA
	Reverse	GCCGTTGAAGTAGAGGGCATT
