# Supplementary material for: Glomerular endothelial cell senescence drives age‐related kidney disease through PAI‐1
Source: EMBO Mol Med. 2021 Nov 2;13(11):e14146. doi: 10.15252/emmm.202114146 (PMC8573606; doi:10.15252/emmm.202114146)
Supplement: Supplementary file 2 — Expanded View Figures PDF [file EMMM-13-e14146-s002.pdf]

Expanded View Figures

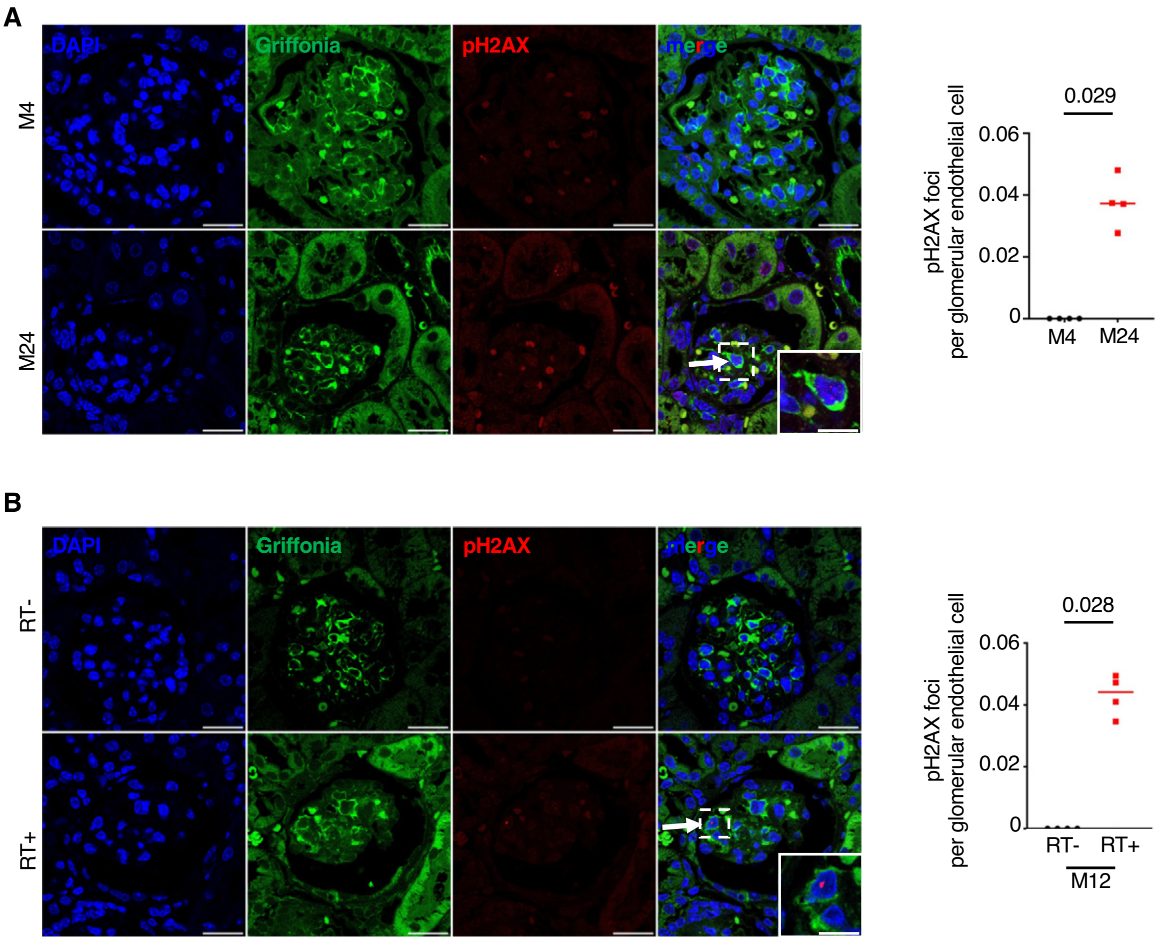

Figure EV1. Senescence affects endothelial cells in glomeruli and increases with age.

A, B pH2AX/griffonia simplicifolia coimmunostaining (left panels) and quantification (right panel) in kidneys from (A) young and aged mice and (B) 12-month-old irradiated and non-irradiated mice. Quantification represents the number of pH2AX foci per glomerular endothelial cell. Original magnification ×630. Scale bar = 10 µm. Panels are representative images of four mice in each group. Data are means ± SEM. Statistical analysis: Student's *t*-test.

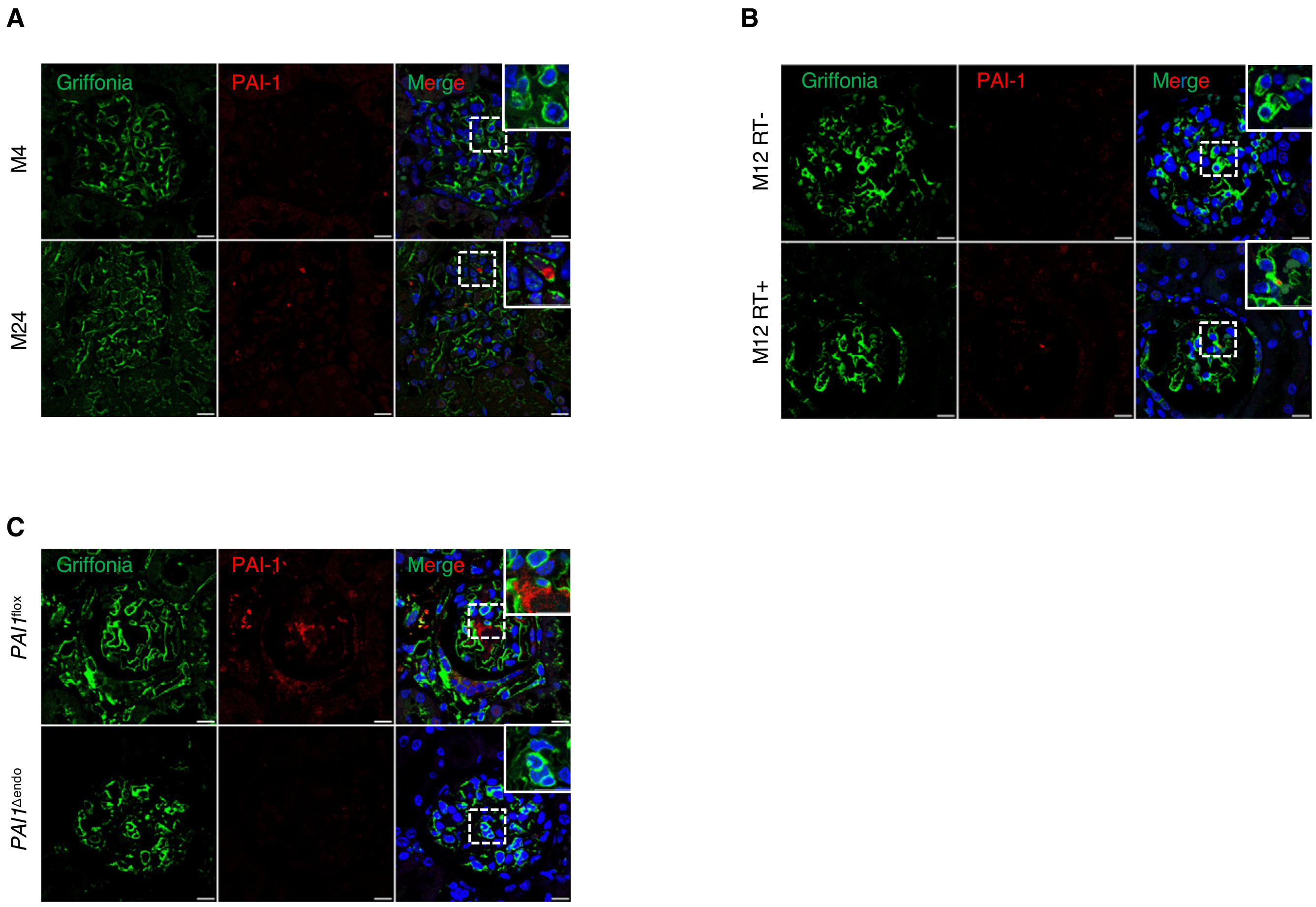

Figure EV2. PAI-1 is expressed in close vicinity to endothelial cells.

A, B PAI-1/griffonia simplicifolia coimmunostaining in kidneys from (A) young and aged mice and (B) 12-month-old irradiated and non-irradiated mice.
C PAI-1/griffonia simplicifolia coimmunostaining in kidneys from 22-month-old *PAI-1*^{flox} and *PAI-1*$^{\Delta endo}$ mice. Original magnification ×630.

Data information: Scale bar = 10 μm. Panels are representative images of four mice in each group.

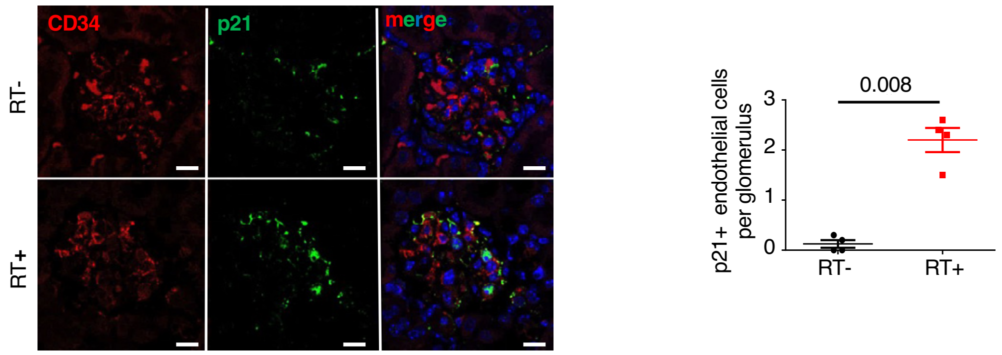

Figure EV3. Senescence affects endothelial cells in glomeruli of irradiated mice.

p21/CD34 coimmunostaining (left panels) and quantification (right panel) in 12-month-old irradiated or non-irradiated mice. Quantification represents the number of p21-positive glomerular endothelial cells. Original magnification ×630. Scale bar = 10 μm. Panels are representative images of four mice in each group. Data are means ± SEM. Statistical analysis: Student's *t*-test.

 © 2021 The Authors

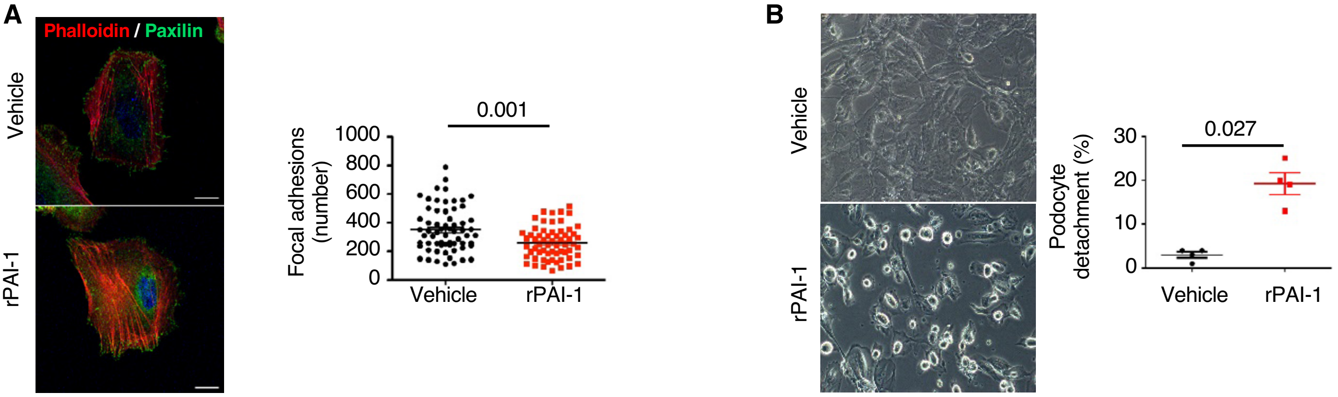

Figure EV4. Recombinant PAI-1 induces podocyte cytoskeleton modifications and detachment.

A Immunofluorescence of paxillin (green) and phalloidin (red) on podocytes stimulated for 30 min with recombinant PAI-1 at 5 nM. Quantification of focal adhesions (right panel) was performed by counting the number of paxillin-positive spots in 30 cells from three independent experiments. Original magnification ×1,000. Scale bar = 20 μm.

B Podocyte morphology (left panels) and quantification of podocyte detachment (right panel) 30 min after stimulation by recombinant PAI-1 at 5 nM. Original magnification ×400, n = 4 independent experiments.

Data information: Data are means ± SEM. Statistical analysis: Student's *t*-test.

© 2021 The Authors
