# Supplementary material for: Glomerular endothelial cell senescence drives age‐related kidney disease through PAI‐1
Source: EMBO Mol Med. 2021 Nov 2;13(11):e14146. doi: 10.15252/emmm.202114146 (PMC8573606; doi:10.15252/emmm.202114146)
Supplement: Supplementary file 3 [file EMMM-13-e14146-s003.pdf]

EMBO Molecular Medicine

Glomerular endothelial cell senescence drives age-related kidney disease through PAI-1

Fabiola Terzi, Camille Cohen, Oceane Le Goff, Frédéric Soysouvanh, Florence Vasseur, Marine Tanou, Clement Nguyen, Lucille Amrouche, Julien Le Guen, Oriana Saltel-Fulero, Tanguy Meunier, Thao Nguyen-Khoa, Marion Rabant, Dominique Nochy, Christophe Legendre, Gerard Friedlander, Bertrand Knebelmann, Dany Anglicheau, Fabien Milliat, Bennett Childs, and Darren Baker
DOI: 10.15252/emmm.202114146

Corresponding author: Fabiola Terzi (fabiola.terzi@inserm.fr)

Review Timeline:

Submission Date:	19th Feb 21
Editorial Decision:	22nd Feb 21
Appeal:	23rd Feb 21
Editorial Decision:	24th Mar 21
Revision Received:	30th Jul 21
Editorial Decision:	1st Sep 21
Revision Received:	16th Sep 21
Accepted:	22nd Sep 21

Editor: Lise Roth

Transaction Report:

(Note: With the exception of the correction of typographical or spelling errors that could be a source of ambiguity, letters and reports are not edited. Depending on transfer agreements, referee reports obtained elsewhere may or may not be included in this compilation. Referee reports are anonymous unless the Referee chooses to sign their reports.)

22nd Feb 2021

Decision on your manuscript EMM-2021-14146

Dear Dr. Terzi,

Thank you for submitting your manuscript "Glomerular endothelial cell senescence drives age-related kidney disease through PAI-1" to EMBO Molecular Medicine. I have now had a chance to discuss your article with the other members of our editorial team. I am afraid that we concluded that your manuscript is not well suited for publication in EMBO Molecular Medicine and have therefore decided not to proceed with peer review.

While potentially of interest to the more immediate community, the article doesn't fit well within EMBO Molecular Medicine as we focus primarily on studies that provide functional insights of translational significance, but also that are conceptually novel and of broad interest. As we do not feel that this is the case here, we therefore cannot offer further consideration to your manuscript.

I am sorry to have to disappoint you on this occasion; in the interest of time, I am providing you with an early decision that will allow you to submit your manuscript elsewhere without any further delays.

Please rest assured that this is not a judgment of the quality or interest of your work, but a decision based on the scope requirement of our journal.

Yours sincerely,

Lise Roth

Lise Roth, PhD
Editor
EMBO Molecular Medicine

As a service to authors, EMBO provides authors with the possibility to transfer a manuscript that one journal cannot offer to publish to another EMBO publication. The full manuscript and if applicable, reviewers reports are automatically sent to the receiving journal to allow for fast handling and a prompt decision on your manuscript. For more details of this service, and to transfer your manuscript to another EMBO title please click on Link Not Available

Please do not share this URL as it will give anyone who clicks it access to your account.

Dear Editor,

Thank you for your rapid reply.

I have understood that the reasons that led the Editorial Board to consider that our manuscript does not deserve to be sent out for a peer reviewing are mainly based on the fact that it is not « conceptually novel and of broad interest ».

I have to confess that I am very surprised by this concern.

On one hand, it is true that PAI-1 is already known to be involved in chronic kidney disease. However, our study is not simply centered on PAI-1. Our study is novel since it sheds a novel light on the pathophysiology of ageing nephropathy. In fact, for the first one, we demonstrated the existence of a cross-talk between senescent endothelial cells and podocytes. It occurs that PAI-1 is merely the messenger of this cross-talk. Hence, mechanistically, our study brings a new vision on how cells communicate insight the kidney in a pathological context.

On the other hand, thanks to a very unique cohort of elderly kidney donors, we have identified the first biomarker susceptible to predict the suitability of kidneys from elderly donors for transplantation. Given the limited availability of grafts, the possibility to rely on kidneys from elderly donors is of an outstanding significant impact in term of public health.

As far as the broad interest, you will agree that elucidating kidney physiopathology is among the scopes of EMBO Mol Med journal, as shown by the number of manuscripts published in this topic. Since 50% of people older than 85 years suffer of kidney disease, we strongly believe that our works will be of great interest not only to scientists working on mechanisms of aging and its role in disease, but also to physician-scientists who try to counteract the deleterious consequences of aging.

Taking all these points into account, I would be very grateful if you will reconsider your decision and give to our manuscript an opportunity to be revised.

I thank you very much for your consideration.

Looking forward to hearing from you,

Sincerely,

Fabiola Terzi

24th Mar 2021

Dear Dr. Terzi,

Thank you for the submission of your manuscript to EMBO Molecular Medicine. We have now received feedback from the three reviewers who agreed to evaluate your manuscript. As you will see from the reports below, the referees acknowledge the interest of the study and are overall supporting publication of your work pending appropriate revisions.

Addressing the reviewers' concerns in full will be necessary for further considering the manuscript in our journal, and acceptance of the manuscript will entail a second round of review. EMBO Molecular Medicine encourages a single round of revision only and therefore, acceptance or rejection of the manuscript will depend on the completeness of your responses included in the next, final version of the manuscript. For this reason, and to save you from any frustrations in the end, I would strongly advise against returning an incomplete revision.

When submitting your revised manuscript, please carefully review the instructions that follow below. Failure to include requested items will delay the evaluation of your revision:

1) A .docx formatted version of the manuscript text (including legends for main figures, EV figures and tables). Please make sure that the changes are highlighted to be clearly visible.

2) Individual production quality figure files as .eps, .tif, .jpg (one file per figure).

3) A .docx formatted letter INCLUDING the reviewers' reports and your detailed point-by-point responses to their comments. As part of the EMBO Press transparent editorial process, the point-by-point response is part of the Review Process File (RPF), which will be published alongside your paper.

4) A complete author checklist, which you can download from our author guidelines (https://www.embopress.org/page/journal/17574684/authorguide#submissionofrevisions). Please insert information in the checklist that is also reflected in the manuscript. The completed author checklist will also be part of the RPF.

5) Before submitting your revision, primary datasets produced in this study need to be deposited in an appropriate public database (see https://www.embopress.org/page/journal/17574684/authorguide#dataavailability).
Please remember to provide a reviewer password if the datasets are not yet public.
The accession numbers and database should be listed in a formal "Data Availability " section (placed after Materials & Method). Please note that the Data Availability Section is restricted to new primary data that are part of this study.
6) We would also encourage you to include the source data for figure panels that show essential data. Numerical data should be provided as individual .xls or .csv files (including a tab describing the data). For blots or microscopy, uncropped images should be submitted (using a zip archive if multiple images need to be supplied for one panel). Additional information on source data and instruction on how to label the files are available at
.

7) Our journal encourages inclusion of *data citations in the reference list* to directly cite datasets that were re-used and obtained from public databases. Data citations in the article text are distinct from normal bibliographical citations and should directly link to the database records from which the data can be accessed. In the main text, data citations are formatted as follows: "Data ref: Smith et al, 2001" or "Data ref: NCBI Sequence Read Archive PRJNA342805, 2017". In the Reference list, data citations must be labeled with "[DATASET]". A data reference must provide the database name, accession number/identifiers and a resolvable link to the landing page from which the data can be accessed at the end of the reference. Further instructions are available at .

8) We replaced Supplementary Information with Expanded View (EV) Figures and Tables that are collapsible/expandable online. A maximum of 5 EV Figures can be typeset. EV Figures should be cited as 'Figure EV1, Figure EV2" etc... in the text and their respective legends should be included in the main text after the legends of regular figures.

- For the figures that you do NOT wish to display as Expanded View figures, they should be bundled together with their legends in a single PDF file called *Appendix*, which should start with a short Table of Content. Appendix figures should be referred to in the main text as: "Appendix Figure S1, Appendix Figure S2" etc.

- Additional Tables/Datasets should be labeled and referred to as Table EV1, Dataset EV1, etc. Legends have to be provided in a separate tab in case of .xls files. Alternatively, the legend can be supplied as a separate text file (README) and zipped together with the Table/Dataset file.

See detailed instructions here:
.

9) The paper explained: EMBO Molecular Medicine articles are accompanied by a summary of the articles to emphasize the major findings in the paper and their medical implications for the non-specialist reader. Please provide a draft summary of your article highlighting
- the medical issue you are addressing,
- the results obtained and
- their clinical impact.

This may be edited to ensure that readers understand the significance and context of the research. Please refer to any of our published articles for an example.

10) For more information: There is space at the end of each article to list relevant web links for further consultation by our readers. Could you identify some relevant ones and provide such information as well? Some examples are patient associations, relevant databases, OMIM/proteins/genes links, author's websites, etc...

11) Every published paper now includes a 'Synopsis' to further enhance discoverability. Synopses are displayed on the journal webpage and are freely accessible to all readers. They include a short stand first (maximum of 300 characters, including space) as well as 2-5 one-sentences bullet points that summarizes the paper. Please write the bullet points to summarize the key NEW findings. They should be designed to be complementary to the abstract - i.e. not repeat the same text. We encourage inclusion of key acronyms and quantitative information (maximum of 30 words / bullet point). Please use the passive voice. Please attach these in a separate file or send them by email, we will incorporate them accordingly.

Please also suggest a striking image or visual abstract to illustrate your article as a png file 550 px-wide x 400-px high.

12) As part of the EMBO Publications transparent editorial process initiative (see our Editorial at http://embomolmed.embopress.org/content/2/9/329), EMBO Molecular Medicine will publish online a Review Process File (RPF) to accompany accepted manuscripts.
In the event of acceptance, this file will be published in conjunction with your paper and will include the anonymous referee reports, your point-by-point response and all pertinent correspondence relating to the manuscript. Let us know whether you agree with the publication of the RPF and as here, if you want to remove or not any figures from it prior to publication.
Please note that the Authors checklist will be published at the end of the RPF.

EMBO Molecular Medicine has a "scooping protection" policy, whereby similar findings that are published by others during review or revision are not a criterion for rejection. Should you decide to submit a revised version, I do ask that you get in touch after three months if you have not completed it, to update us on the status.

I look forward to receiving your revised manuscript.

Yours sincerely,

Lise Roth

Lise Roth, PhD
Editor
EMBO Molecular Medicine

***** Reviewer's comments *****

Referee #1 (Comments on Novelty/Model System for Author):

In this work, Cohen et al investigate the role of cellular senescence in the kidney during glomerulosclerosis in the context of aging using cells and mouse models, as well as human samples. They observed in old or irradiated mice accumulation of senescent cells, probably mainly endothelial cells, in the glomerulus, which correlates to glomerulosclerosis, decrease podocytes numbers and apoptosis. Their results, mainly through the use of mice specifically depleted in PAI1 in endothelial cells, support that PAI1, a SASP factor, participates to the above described alterations in the glomeruli during aging. In addition, in vitro data using supernatant of senescent (replicative) primary glomerular endothelial cells, with or without PAI1 inhibition, or using PAI1 recombinant protein, on immortalized human podocytes confirm a role of PAI1 in regulating podocytes detachment. To finish, the authors display results on human samples showing a correlation between PAI1 levels and cellular senescence in glomeruli of old individuals. The experiments are globally well designed and convincing and support the overall message of the authors. The message sounds novel and relevant for EMBO Mol Med journal. Nevertheless, some limits and issues should be addressed before potential publication:

-when senescence is explored in the Figures 1-3, it will be good in addition of analyzing senescence in glomeruli to also examine cellular senescence and PAI1 production in the other parts of the kidney such as in tubules. mRNA data on SASP factors seem to be done on whole kidney samples and not micro dissected glomeruli and are thus not that informative except if senescence occurs only/mainly in the glomeruli during aging or after irradiation.
-some of the images/data/graphs are not that convincing: Figure 1D SA-bGal images is not convincing and should be replaced; 53BP1 staining with 1 dot per nuclei is rather low, can these results be confirmed by another DNA damage marker such as gH2AX; quantification of 53BP1 is shown as 53BP1 spots/nm2, it should be replaced by 53BP1 spots per cell or percentage of cells with or without spots (eventually in CD34+ and - population see comments below).
-as an important message of the author rely on the fact that senescent endothelial cells produce PAI1, this should be reinforced by for example performing costaining between markers of senescence and markers of endothelial cells (VE-cadh could be added for example). So far the only costaining is between CD34 (used a marker of endothelial cells) and 53BP1 (used) as a marker of senescent cells. Quantification is performed for 53BP1, could be good to do it also for p21 after costaining with CD34, to quantify the percentage of positive cells (53BP1, p21) in the CD34+ cells and CD34- cells inside the glomeruli.
-in Figure 1C it will be good to show that it is the podocytes that are dying
-in Figure 4, can the author show the level of PAI1 in senescent endothelial cells and use other classical senescence markers (p21, p16 and some other SASP factors ?); is it possible to measure other parameters on podocytes to evaluate their loss of function beyond detachment, for instance cell death ?
-globally the authors should carefully modify their manuscript to avoid overinterpretation of their results such as the titles used in their results which are not fully supported by the data shown in the commented figure, avoid overinterpreting mRNA levels in the glomeruli if what is measured is in total kidney...
-in Figure 5 on human samples: will be good to co-stain senescent cells and endothelial cells as done in mouse samples (Figure 5B); will be good to stain samples used in Figure 5D&E against a senescence marker such as p16; can the authors comment about urinary PAI1 levels compared to urinary level of other SASP factors during CKD.

Referee #2 (Comments on Novelty/Model System for Author):

As stated in my report, I have some concerns about the level of novelty of this study. However, I think the hypothesis is interesting and the human data together with the preclinical data with mouse models could represent a significant research contribution. The field of senescence and how it relates to multiple age-related disorders is a hot topic, and the identification of a potential biomarker for kidney dysfunction justifies the interest of the study.

Referee #2 (Remarks for Author):

In this manuscript entitled "Glomerular endothelial cell senescence drives age-related kidney disease through PAI-1", Cohen et al. propose a detrimental cross-talk between senescent endothelial cells and podocytes driven by PAI-1. The authors show evidence that the selective inactivation of PAI-1 in endothelial cells protected glomeruli from the development of lesions and podocyte loss in aged mice. In addition, blocking PAI-1 in supernatants from senescent endothelial cells seems to prevent podocyte detachment and dedifferentiation in cultures. Finally, the authors suggest the experimental findings are relevant

findings are relevant to humans and that glomerular PAI-1 expression predicts poor outcomes of transplanted kidneys from elderly donors. The use of PAI-1 as a prognostic marker and potential therapeutic target is further supported by the association of urinary PAI-1 in elderly patients with age-related chronic kidney disease.

Overall, the manuscript is well written and organized, but the data do not support some of the claims of the authors at this stage and I have some concerns regarding the novelty of this study. I consider there are major concerns as outlined below that should be addressed before recommending this article for its publication in EMBO Mol Med.

Major concerns:

1. As indicated by the authors, the association between PAI-1 expression and the development of glomerular lesions in several experimental models of kidney and glomerular diseases is well-known and has been reported in a number of studies from many years ago (Paueksakon et al. 2002, Yamamoto et al. 1993, Hamano et al. 2002, etc.). Also, PAI-1 can serve as both a marker and a promoter or mediator of cell senescence, and it has been shown to be part of the SASP and contribute to detrimental roles in senescence-related disorders, such as fibrogenic activities, atherogenesis, obesity, diabetes, emphysema and even major depressive disorders (Vaughan et al. 2017). Moreover, PAI-1 governs senescence and its levels are significantly elevated in Klotho hypomorphic mice (Eren et al. 2014), and depletion of senescent cells ameliorates glomerulosclerosis in aged mice (Baker et al. 2016). Altogether, the authors should clearly clarify and justify the level of novelty of this study and highlight the aspect/s they consider represent a real advance in the field or at the preclinical level and, conversely, recognize the limitations of this work, when appropriate.

2. In my opinion, the authors exceed their claims as they overinterpret the causal connection between the implementation of senescence and the accumulation of PAI-1. Here, the existing data do not (at least completely) support the conclusions of the authors. From the existing results, it cannot be excluded just a circumstantial association between senescence and PAI-1. I find crucial to show that, upon senolytic treatment (e.g. dasatinib + quercetin, ABT-263, etc.), PAI-1 does not accumulate in the glomerular lesions and, if the hypothesis of the authors is correct, this should result in less podocytes death and reduction in the serum creatinine. Alternatively, the authors might use a genetic model to ablate p16-positive cells (INK-ATTAC or p16.3MR mice, or one of the models published last year in Cell Metabolism) but I think the senolytic approach should be compelling.

3. Following the above, I think the assessment of senescence throughout the paper is sometimes weak, the SAB-gal activity in tissues is not easy to measure. As an example, in Figure 1 there is evidence of colocalization between CD34- and 53BP1-positive cells but not in the case of p21. 53BP1 is a maker of damage but not necessarily a senescent marker. Also, quantifications in double IFs are missing in Figures 1-3. More robust evidence of senescence in endothelial cells needs to be presented. Again, the authors cannot exclude the implementation of senescence in other cell types. Have they tried to perform colocalization studies of podocytes or other glomerular cell types with markers of senescence (e.g. p21)?

4. The results provided in Figure 4 are stimulating but the authors do not provide any mechanistic insights in how endothelial PAI-1 induces podocyte detachment involving cytoskeleton reorganization. Again, what happens if the supernatants are added to other cell types? A more convincing experiment would be to co-culture senescent endothelial cells with podocytes, for example senescent endothelial cells expressing mCherry and podocytes expressing GFP, and to show that podocytes detach from the plates but not when co-cultured with normal endothelial cells. In addition, a crucial control in this experiment is missing, as the authors do not show evidence of PAI-1 inhibition upon tiplaxtinin exposure.

5. The data presented in Figure 5 are interesting and a remarkable support to the authors´ hypothesis. However, I´m confused on how quantifications in Figures 5A-B and Tables were done. Instead of percentage of patients with PAI-1+ and p16+ glomeruli it had been better to show the percentages of PAI-1+ and p16+ glomeruli per patient. I mean, among the 18 donors showing glomerular PAI-1 staining at time of transplantation what is the percentage of PAI-1 and p16-positive glomeruli? Again, I think the assessment of senescence in patient´s samples is weak. Evidence of other markers such as p21 would reinforce the conclusions and, most importantly, double-staining with CD34. It is important to provide evidence of endothelial senescence with patients' samples.

Other points/suggestions:
1. A compelling experiment would be to microdissect glomeruli from either the mouse models or the patients´samples and perform RNAseq analyses. This would provide key information on the upregulation of pathways related to cellular senescence and SASP, and it might shed some light on the underlying mechanisms. One would expect that PAI-1 is a differentially expressed gene in aged or irradiated mice, or in samples from old donors

2. The quality of histological images in some Figures is poor. Magnifications of key areas and scales should be included in all of the figures.

Referee #3 (Remarks for Author):

Cohen and colleagues examine the crosstalk between senescent endothelial cells and podocytes in the development of glomerular lesions during aging. Old mice and mice exposed to radiation present with senescent endothelial cells in the glomerulus which is associated with elevated PAI-1 and podocyte apoptosis. The authors show deletion of PAI-1 in endothelial cells is protective against glomerular lesions and podocyte loss in aged mice. In cultured podocytes exposed to supernatants from senescent endothelial cells, podocytes detach, and this is prevented by PAI-1 blockade. Finally, in human samples, glomerular PAI-1 predicted poor outcome of transplanted kidneys from elderly donors and urinary PAI-1 was associated with age-related chronic kidney disease. Collectively, this is an interesting study with a high degree of novelty. We know that podocytes release molecules that modulate the glomerular endothelium, but there is little information on molecular communication in the other direction. A strength of the article is the use of multiple strategies in animals, cells, and human samples. However, there are some issues to address. In particular, the data showing that senescence cells are endothelial should be strengthened. Additionally, the authors should use additional time-points to tease out whether the presence of senescence cells occurs prior to podocyte apoptosis.

Major Comments

1. The authors first examine podocyte apoptosis and senescence in the glomeruli of 4 and 24-month-old mice. There are a few issues to be resolved here:
(i) It is stated that senescence cells were identified mainly in glomeruli. This is rather a vague statement, were senescence cells also seen in other area of the kidney;
(ii) Were p16 positive cells also seen in the glomerulus? Can double labelling be performed to show multiple senescence markers are expressed on the same cells?
(iii) The reviewer would like to see stronger evidence that the senescence cells were found in the endothelium. Double labelling is performed with 53BP1 and CD34, but can other senescence markers be used for staining or FACS analysis. Can the authors show that the senescence cells do not express markers of other glomerular cell types?
(iv) Are there changes in renal function between the 4 and 24-month-old mice?

2. A key question that remains from Figure 1 (and the remainder of the work) is whether the senescence glomerular endothelial cells are driving podocyte apoptosis. The authors only compare 4-month-old mice with 24-month-old mice, a large difference in age. The article would be strengthened if intermediate time-points were used, this may allow the authors to tease out if senescence occurs early than podocyte apoptosis.

3. What was the rationale for focusing on PAI-1, IL-1b, IL-6 and MMP-13 as SASP components? Why was it surprising that VEGF-A was downregulated? The authors show that PAI-1 is upregulated in glomeruli, but can this be pinpointed to endothelial cells.

4. Similar questions outlined in points 1-3 should be addressed in the set of experiments where irradiation is used to induce senescence and glomerular lesions.

5. A strategy is then taken to delete PAI-1 in the endothelium using VE-cadherin Cre. A key control is missing, animals with Cre alone, do the authors have data on this as Cre recombinase can itself promote glomerular injury (Balkawade Am J Physiol Renal Physiol 2019 316: F1026-40). VE-cadherin Cre will delete vessels in the whole-body of the mouse not just the kidney so was there any phenotype in other organs. One time-point is examined (at 22 weeks) so only an association between senescence and podocyte apoptosis can be made. As highlighted above the paper would be strengthened if multiple time-points had been examined.

6. Cell culture is used to examine endothelial-podocyte crosstalk in more detail. Primary glomerular endothelial cells are used - can the authors show they express appropriate markers and if they are maintained as passage number increases. Can quantification of the actin cytoskeleton be performed once endothelial cell supernatants have been added to podocytes? In these experiments, 30 cells from 3 independent experiments are assessed for focal adhesions. The data is presented for each cell, but are there also differences if the means of each experiment are plotted. Is there apoptosis of the podocyte as seen in vitro?

7. The role of PAI-1 in endothelial-podocyte crosstalk in culture is examined. Do the authors know if PAI-1 secretion is increased as the number of passages the endothelial cells undertake increase? Does Tiplaxtinin reduce the levels of PAI-1 in the supernatant? The effect on podocyte apoptosis should be evaluated.

8. PAI-1 is then examined in young and old transplant donors, with increased glomerular expression in the older donors. Can the authors pinpoint expression to the endothelium?

9.page 14, 2nd paragraph. It seems rather strong to suggest that PAI-1 is the mediator of the detrimental crosstalk between endothelial cells and podocytes. It is likely other mechanisms are involved. Similarly, bottom paragraph of page 15, we do not know if PAI-1 is the critical mediator in this process.

Minor Comments:

1. page 3, 2nd paragraph. The authors give an example of changes in glomerulosclerosis in aging but the comparison is dramatic between 18-29 years old and 70+. Is there any evidence of a step wise increase in glomerulosclerosis with increasing

age?

2. page 3. An article by Baker et al, 2016 is presented as an example that senescent cells are involved in glomerulosclerosis but can more detail by provided. At the moment the paper just mentions it is a very elegant model. Please expand.

3. page 4, end of 2nd paragraph. Rather vague. Please add details of which SASP molecules play a role in the development of glomerular lesions.

Ref: EMM-2021-14146

Point-by-point responses to reviewer's comments

We thank the three Reviewers for their thoughtful comments. We feel that the revised version of the manuscript has been considerably improved by their inputs/criticisms.

Referee #1

In this work, Cohen et al investigate the role of cellular senescence in the kidney during glomerulosclerosis in the context of aging using cells and mouse models, as well as human samples. They observed in old or irradiated mice accumulation of senescent cells, probably mainly endothelial cells, in the glomerulus, which correlates to glomerulosclerosis, decrease podocytes numbers and apoptosis. Their results, mainly through the use of mice specifically depleted in PAI1 in endothelial cells, support that PAI1, a SASP factor, participates to the above described alterations in the glomeruli during aging. In addition, in vitro data using supernatant of senescent (replicative) primary glomerular endothelial cells, with or without PAI1 inhibition, or using PAI1 recombinant protein, on immortalized human podocytes confirm a role of PAI1 in regulating podocytes detachment. To finish, the authors display results on human samples showing a correlation between PAI1 levels and cellular senescence in glomeruli of old individuals. The experiments are globally well designed and convincing and support the overall message of the authors. The message sounds novel and relevant for EMBO Mol Med journal. Nevertheless, some limits and issues should be addressed before potential publication:

-when senescence is explored in the Figures 1-3, it will be good in addition of analyzing senescence in glomeruli to also examine cellular senescence and PAI1 production in the other parts of the kidney such as in tubules. mRNA data on SASP factors seem to be done on whole kidney samples and not micro dissected glomeruli and are thus not that informative except if senescence occurs only/mainly in the glomeruli during aging or after irradiation.

We agree with the Reviewer that, consistent with previous studies (*Bhardwaj G et al, Nature 2016, 538:329-335; Omori S et al, Cell Metab 2020, 32:814-828; Melk A et al, Kidney Int 2004, 65:510-520),* senescence might affect tubular cells. Hence, we performed additional experiments aimed at quantifying the extent of senescence in all the kidney sections over time in aging and irradiated mice. Results showed that p21 staining increased in both glomerular and tubular cells from 18 months of age, and as soon as 4 months after irradiation. Interestingly, in the tubulo-interstitial compartment, p21 was expressed exclusively in tubular cells, whereas co-staining experiments clearly showed that p21 was mainly expressed in endothelial cells in glomeruli (see Figure 1). These results are now shown in the new Figure 3.

-some of the images/data/graphs are not that convincing: Figure 1D SA-bGal images is not convincing and should be replaced;

We apologize for the quality of the images in Figure 1D. They have been replaced in the new version of the manuscript.

53BP1 staining with 1 dot per nuclei is rather low, can these results be confirmed by another DNA damage marker such as gH2AX; quantification of 53BP1 is shown as 53BP1 spots/nm2, it should be replaced by 53BP1 spots per cell or percentage of cells with or without spots (eventually in CD34+ and - population see comments below).

As requested, we performed co-immunostaining experiments using pH2AX antibodies and griffonia simplicifolia, a lectin that specifically recognizes endothelial cells, in kidneys of aging and irradiated mice. The quantification of pH2AX foci confirmed the increase of DNA damage in glomerular endothelial cells with age and irradiation. The new data are shown in the new Figure EV1.

In addition, 53BP1 staining was re-quantified and the data are now expressed as the number of foci per glomerular endothelial nuclei in the new Figure 1F and new Figure 2F.

-as an important message of the author rely on the fact that senescent endothelial cells produce PAI1, this should be reinforced by for example performing costaining between markers of senescence and markers of endothelial cells (VE-cadh could be added for example). So far the only costaining is between CD34 (used a marker of endothelial cells) and 53BP1 (used) as a marker of senescent cells. Quantification is performed for 53BP1, could be good to do it also for p21 after costaining with CD34, to quantify the percentage of positive cells (53BP1, p21) in the CD34+ cells and CD34- cells inside the glomeruli.

We greatly appreciate this suggestion. As requested, we carried out several co-immunostaining experiments, which confirmed that senescence affects mainly endothelial cells in glomeruli. In particular, we performed triple co-immunofluorescence experiments using antibodies directed against p21, nephrin (a marker of podocytes), and the lectin griffonia. Results confirmed that p21 co-localizes with griffonia, but not with nephrin in both aged and irradiated mice (see new Figure 1E and new Figure 2E). In addition, we confirmed that p21 colocalizes with CD34 in endothelial glomerular cells of irradiated mice (see new Figure EV3).

It is worth noting that a recent paper also reported that endothelial cells may undergo cellular senescence in different physiological and pathological contexts (*Omori S et al, Cell Metab 2020, 32:814-828*). These findings are discussed in the revised version of the manuscript.

-in Figure 1C it will be good to show that it is the podocytes that are dying

As requested, we have tried to show *in vivo* that apoptosis affects podocytes by performing several co-immunostaining experiments using TUNEL or cleaved caspase-3 antibodies to detect apoptosis and nephrin or WT1 to detect podocytes. Unfortunately, we unable to obtain experimental conditions that allowed for suitable co-staining. In fact, we always obtained either an absence of staining or a high background. That said, as requested by Reviewers 2 and 3, we investigated *in vitro* whether supernatants from senescent endothelial cells trigger podocyte apoptosis in co-culture experiments. Results clearly showed that this was the case. The new data are shown in the new Figure 6G.

-in Figure 4, can the author show the level of PAI1 in senescent endothelial cells and use other classical senescence markers (p21, p16 and some other SASP factors ?); is it possible to measure other parameters on podocytes to evaluate their loss of function beyond detachment, for instance cell death ?

We thank the reviewer for this suggestion. As requested, we measured p21 and p16 mRNA expression levels, as markers of senescence; and PAI-1, IL-6 and IL-8, as markers of SASP in primary glomerular endothelial cells. Results clearly showed that all these markers were significantly increased in senescent cells from late passages (P17) as compared to non-senescent cells from early passages (P9). These data are shown in the new Figure 6C and 6D.

As indicated above, apoptosis has now been studied in podocytes incubated with supernatants from senescent endothelial cells.

-globally the authors should carefully modify their manuscript to avoid overinterpretation of their results such as the titles used in their results which are not fully supported by the data shown in the commented figure, avoid overinterpreting mRNA levels in the glomeruli if what is measured is in total kidney...

We thank the reviewers for this comment. The manuscript has been revised taking into account this point.

-in Figure 5 on human samples: will be good to co-stain senescent cells and endothelial cells as done in mouse samples (Figure 5B); will be good to stain samples used in Figure 5D&E against a senescence marker such as p16; can the authors comment about urinary PAI1 levels compared to urinary level of other SASP factors during CKD.

As requested, we performed co-immunostaining experiments on biopsies from elderly donors using antibodies directed against p16 and CD34. Results clearly showed that p16 colocalizes with CD34. We also did co-immunostaining with PAI-1 and CD34 antibodies. Similarly, results indicated that PAI-1, a secreted factor, is expressed in close vicinity to CD34. The fact that PAI-1 is a secreted protein may account for not perfectly overlapping with griffonia/CD34 staining. These results are illustrated in the new Figures 7D and 7C, respectively.

It is true that PAI1 is not the only SASP molecule secreted in the urine of CKD patients. For example, it has been reported that the excretion of IL6 (*Wolkow PP et al, J Am Soc Nep 2008, 19:789-797; Lipiec K et al, Adv Clin Exp Med 2018, 27:955-962*), IL18 (*Bullen AL, Am J Kidney Dis 2021, doi: 10.1053/j.ajkd.2021.01.021*), or MMP9 (*Musial K et al, Biomarkers 2015, 20:177-182598*) are also increased during CKD. Nevertheless, since none of the SASP molecules are specific of senescence, it is difficult to assess if the secretion of these factors is really due to senescence or to a more general process involved in CKD progression. This is now better discussed in the revised version of the manuscript and the references are quoted.

Referee #2

As stated in my report, I have some concerns about the level of novelty of this study. However, I think the hypothesis is interesting and the human data together with the preclinical data with mouse models could represent a significant research contribution. The field of senescence and how it relates to multiple age-related disorders is a hot topic, and the identification of a potential biomarker for kidney dysfunction justifies the interest of the study.

In this manuscript entitled "Glomerular endothelial cell senescence drives age-related kidney disease through PAI-1", Cohen et al. propose a detrimental cross-talk between senescent endothelial cells and podocytes driven by PAI-1. The authors show evidence that the selective inactivation of PAI-1 in endothelial cells protected glomeruli from the development of lesions and podocyte loss in aged mice. In addition, blocking PAI-1 in supernatants from senescent endothelial cells seems to prevent podocyte detachment and dedifferentiation in cultures. Finally, the authors suggest the experimental findings are relevant findings are relevant to humans and that glomerular PAI-1 expression predicts poor outcomes of transplanted kidneys from elderly donors. The use of PAI-1 as a prognostic marker and potential therapeutic target is further supported by the association of urinary PAI-1 in elderly patients with age-related chronic kidney disease.

Overall, the manuscript is well written and organized, but the data do not support some of the claims of the authors at this stage and I have some concerns regarding the novelty of this study. I consider there are major concerns as outlined below that should be addressed before recommending this article for its publication in EMBO Mol Med.

Major concerns:

1. As indicated by the authors, the association between PAI-1 expression and the development of glomerular lesions in several experimental models of kidney and glomerular diseases is well-known and has been reported in a number of studies from many years ago (Paueksakon et al. 2002, Yamamoto et al. 1993, Hamano et al. 2002, etc.). Also, PAI-1 can serve as both a marker and a promoter or mediator of cell senescence, and it has been shown to be part of the SASP and contribute to detrimental roles in senescence-related disorders, such as fibrogenic activities, atherogenesis, obesity, diabetes, emphysema and even major depressive disorders (Vaughan et al. 2017). Moreover, PAI-1 governs senescence and its levels are significantly elevated in Klotho hypomorphic mice (Eren et al. 2014), and depletion of senescent cells ameliorates glomerulosclerosis in aged mice (Baker et al. 2016). Altogether, the authors should clearly clarify and justify the level of novelty of this study and highlight the aspect/s they consider represent a real advance in the field or at the preclinical level and, conversely, recognize the limitations of this work, when appropriate.

We agree with the Reviewer that PAI-1 has already been associated with kidney disease. In fact, we highlight this point in the discussion section of our manuscript. It is also true that Baker et al. has elegantly showed that depletion of senescent cells ameliorates glomerulosclerosis in aged mice. However, we believe that our study brings real novel mechanistic insights into the pathogenesis of aging nephropathy. We show, for the first time, an endothelial – podocyte cross-talk that is detrimental for podocyte survival with age. The finding that senescence affects glomerular endothelial cells is, to the best of our knowledge, also a new finding. More importantly, we have identified a novel biomarker able to predict the survival of kidney allografts from elderly donors, a critical issue for medical care. These points are now better highlighted in the revised version of the manuscript.

2. In my opinion, the authors exceed their claims as they overinterpret the causal connection between the implementation of senescence and the accumulation of PAI-1. Here, the existing data do not (at least completely) support the conclusions of the authors. From the existing results, it cannot be excluded just a circumstantial association between senescence and PAI-1. I find crucial to show that, upon senolytic treatment (e.g. dasatinib + quercetin, ABT-263, etc.), PAI-1 does not accumulate in the glomerular lesions and, if the hypothesis of the authors is correct, this should result in less podocytes death and reduction in the serum creatinine. Alternatively, the authors might use a genetic model to ablate p16-positive cells (INK-ATTAC or p16.3MR mice, or one of the models published last year in Cell Metabolism) but I think the senolytic approach should be compelling.

We thank the reviewer for raising this important point. Following this suggestion, we collaborated with Dr Baker in order to determine whether depletion of senescent cells affects podocyte survival and glomerular PAI-1 expression by using the *p16 INK-ATTAC* mouse model. As the Reviewer knows, this transgenic model is characterized by the clearance of p16 positive cells after injection of AP20187. We confirmed that glomerulosclerosis was reduced in aged transgenic mice as compared to controls. More importantly, we demonstrated that the beneficial effect of glomerular senescent cell depletion was associated with an increased number of podocytes, whereas PAI-1 expression was decreased. These data are shown in the new Figure 4.

3. Following the above, I think the assessment of senescence throughout the paper is sometimes weak, the SAB-gal activity in tissues is not easy to measure. As an example, in Figure 1 there is evidence of colocalization between CD34- and 53BP1-positive cells but not in the case of p21. 53BP1 is a maker of damage but not necessarily a senescent marker. Also, quantifications in double IFs are missing in Figures 1-3. More robust evidence of senescence in endothelial cells needs to be presented. Again, the authors cannot exclude the

implementation of senescence in other cell types. Have they tried to perform colocalization studies of podocytes or other glomerular cell types with markers of senescence (e.g. p21)?

To reinforce our data on the occurrence of senescence in glomerular endothelial cells, we carried out several co-immunostaining experiments. In particular, we performed a triple co-immunofluorescence experiment using antibodies directed against p21, nephrin (a marker of podocytes), and the lectin griffonia simplicifolia, as a specific endothelial marker. Results confirmed that p21 co-localizes with griffonia, but not with nephrin in both aged and irradiated mice (see new Figure 1E and new Figure 2E). In addition, we confirmed that p21 colocalizes with CD34 in endothelial glomerular cells of irradiated mice (see new Figure EV3).

It is worth noting that a recent paper also reported that endothelial cells may undergo cellular senescence in different physiological and pathological contexts (*Omori S et al, Cell Metab 2020, 32:814-828*). These findings have been discussed in the revised version of the manuscript.

4. The results provided in Figure 4 are stimulating but the authors do not provide any mechanistic insights in how endothelial PAI-1 induces podocyte detachment involving cytoskeleton reorganization. Again, what happens if the supernatants are added to other cell types? A more convincing experiment would be to co-culture senescent endothelial cells with podocytes, for example senescent endothelial cells expressing mCherry and podocytes expressing GFP, and to show that podocytes detach from the plates but not when co-cultured with normal endothelial cells. In addition, a crucial control in this experiment is missing, as the authors do not show evidence of PAI-1 inhibition upon tiplaxtinin exposure.

To provide further mechanistic insights on the cross-talk between senescent endothelial cells and podocytes, we analyzed podocyte apoptosis after incubation with supernatants from endothelial cells at early (P9) or late (P17) passage in the presence, or not, of Tiplaxtinin by performing FACS experiments with Annexin-V. Interestingly, we observed an increase of apoptosis in podocytes cultured with late passage (senescent) supernatants as compared to early passage supernatants. More importantly, the increase was prevented by incubation with Tiplaxtinin. These data are shown in the new Figure 6G.

In order to investigate whether Tiplaxtinin inhibits PAI-1, we attempted reverse zymography of supernatants with or without Tiplaxtinin. We chose this approach since Tiplaxtinin is known to inhibit the enzymatic activity of PAI-1. Although we succeeded in showing that recombinant PAI-1 activity was inhibited by Tiplaxtinin, technical problems prevented us from reaching any conclusions. In fact, incubation of the gels with the supernatants from endothelial cells resulted in a strong background (see Reviewer Figure 1). We believe that this is due to the serum contained in the supernatant.

5. The data presented in Figure 5 are interesting and a remarkable support to the authors´ hypothesis. However, I´m confused on how quantifications in Figures 5A-B and Tables were done. Instead of percentage of patients with PAI-1+ and p16+ glomeruli it had been better to show the percentages of PAI-1+ and p16+ glomeruli per patient. I mean, among the 18 donors showing glomerular PAI-1 staining at time of transplantation what is the percentage of PAI-1 and p16-positive glomeruli? Again, I think the assessment of senescence in patient´s samples is weak. Evidence of other markers such as p21 would reinforce the conclusions and, most importantly, double-staining with CD34. It is important to provide evidence of endothelial senescence with patients' samples.

As requested, the percentages of PAI-1+ and p16+ glomeruli per patient have now been quantified and are present in the new Figure 7A and 7B, respectively.

We have also carried out additional experiments to reinforce our human data. In particular, we performed co-immunostaining experiments on biopsies from elderly donors using antibodies directed against p16 and CD34. Results clearly showed that p16 colocalizes with CD34. We also did co-immunostaining with PAI-1 and CD34 antibodies. Similarly, the results indicate that PAI-1, a secreted factor, is expressed in close vicinity to CD34. The fact that PAI-1 is a secreted protein may account for not perfectly overlapping with griffonia/CD34 staining. These results are illustrated in the new Figure 7D and 7C. For p21 immunostaining, we tested several experimental conditions but unfortunately, were unable to detect a specific positive signal in alcohol-formalin–acetic acid solution fixed human biopsies.

Other points/suggestions:

1. A compelling experiment would be to microdissect glomeruli from either the mouse models or the patients´samples and perform RNAseq analyses. This would provide key information on the upregulation of pathways related to cellular senescence and SASP, and it might shed some light on the underlying mechanisms. One would expect that PAI-1 is a differentially expressed gene in aged or irradiated mice, or in samples from old donors

We agree with the reviewer that it would be of great interest to perform RNA-seq analyses on microdissected glomeruli. Nevertheless, this was out of the scope of the present study.

2. The quality of histological images in some Figures is poor. Magnifications of key areas and scales should be included in all of the figures.

We apologize for the poor quality of some images. This has been improved in the revised version of the manuscript and magnifications and scales have now been added to all the Figures.

Referee #3

Cohen and colleagues examine the crosstalk between senescent endothelial cells and podocytes in the development of glomerular lesions during aging. Old mice and mice exposed to radiation present with senescent endothelial cells in the glomerulus which is associated with elevated PAI-1 and podocyte apoptosis. The authors show deletion of PAI-1 in endothelial cells is protective against glomerular lesions and podocyte loss in aged mice. In cultured podocytes exposed to supernatants from senescent endothelial cells, podocytes detach, and this is prevented by PAI-1 blockade. Finally, in human samples, glomerular PAI-1 predicted poor outcome of transplanted kidneys from elderly donors and urinary PAI-1 was associated with age-related chronic kidney disease. Collectively, this is an interesting study with a high degree of novelty. We know that podocytes release molecules that modulate the glomerular endothelium, but there is little information on molecular communication in the other direction. A strength of the article is the use of multiple strategies in animals, cells, and human samples. However, there are some issues to address. In particular, the data showing that senescence cells are endothelial should be strengthened. Additionally, the authors should use additional time-points to tease out whether the presence of senescence cells occurs prior to podocyte apoptosis.

Major Comments

1. The authors first examine podocyte apoptosis and senescence in the glomeruli of 4 and 24-month-old mice. There are a few issues to be resolved here:

(i) It is stated that senescence cells were identified mainly in glomeruli. This is rather a vague statement, were senescence cells also seen in other area of the kidney;

We thank the reviewer for raising this important point. As discussed above, we performed additional experiments aimed at quantifying p21 staining, a marker of cellular senescence, over time in tubular sections of aging and irradiated mice. Results clearly showed that senescence increased in both glomerular and tubular cells from 18 months of age, and as soon as 4 months after irradiation. Interestingly, in the tubulo-interstitial compartment, p21 was expressed exclusively in tubular cells, whereas co-staining experiments clearly showed that p21 was mainly expressed in endothelial cells of glomeruli (see Figure 1). These results are now shown in the new Figure 3.

(ii) Were p16 positive cells also seen in the glomerulus? Can double labelling be performed to show multiple senescence markers are expressed on the same cells?

To definitively prove that p16 is expressed in glomerular endothelial cells, we carried out co-immunostaining experiments with p16 and CD34 antibodies. Results showed that p16 colocalizes with CD34 in human biopsies. Similarly, by performing a triple co-immunostaining with p21, nephrin (a marker of podocytes) and griffonia simplicifolia (a lectin directed against endothelial cells) or a double co-immunostaining with 53BP1 and CD34, we demonstrated that p21 and 53BP1 are expressed in endothelial cells (see new Figure 1E, 1F, 2E, 2F). In addition, we confirmed that p21 colocalizes with CD34 in endothelial glomerular cells of irradiated mice (see new Figure EV3). Altogether, these data clearly indicate that senescence affects endothelial glomerular cells.

(iii) The reviewer would like to see stronger evidence that the senescence cells were found in the endothelium. Double labelling is performed with 53BP1 and CD34, but can other senescence markers be used for staining or FACS analysis. Can the authors show that the senescence cells do not express markers of other glomerular cell types?

As indicated above, in the revised version of the manuscript, we have considerably reinforced the results showing that senescence in glomeruli affects endothelial cells. In both aged and irradiated mice, we performed triple co-immunofluorescence with p21, nephrin and griffonia. Results clearly show that p21 colocalizes with griffonia, but not with nephrin. In addition, we carried out p21 and CD34 co-immunofluorescence experiments and confirmed the endothelial expression of p21 in glomeruli of irradiated mice.

(iv) Are there changes in renal function between the 4 and 24-month-old mice?

Unfortunately, we did not collect blood in this study, since we did not have the ethical agreement. However, we measured serum creatinine in 24-month-old $PAI\text{-}1^{\Delta endo}$ mice and in counterpart littermates. Results demonstrated that renal function was significantly improved by endothelial PAI-1 deletion (see Figure 5E). This is consistent with an impairment in renal function from 4 to 24 months. It is worth noting that serum creatinine is usually around 10 μM in young mice, but it was double in the 24-month-old wild-type mice.

2. A key question that remains from Figure 1 (and the remainder of the work) is whether the senescence glomerular endothelial cells are driving podocyte apoptosis. The authors only compare 4-month-old mice with 24-month-old mice, a large difference in age. The article would be strengthened if intermediate time-points were used, this may allow the authors to tease out if senescence occurs early than podocyte apoptosis.

We very much appreciate this suggestion. As requested, we carried out complementary experiments to better characterize the evolution of senescence over time. First, we studied p21 expression in tubules and glomeruli at 4, 12, 18 and 24 months of age. We observed that senescence appeared at 18 months in both tubular and glomerular cells. A time course analysis of WT-1 expression demonstrated that podocyte numbers began to decrease at the

same time point (18 months). In addition, we observed that p21 expression progressively increased from 4 to 12 months in irradiated mice. Podocyte decrease followed the same trend. To go further, we studied a transgenic model of mice expressing luciferase under the control of the p16 promoter. A bioluminescence assay performed over time confirmed that senescence appeared at 18 months in kidneys of aging mice. In contrast, in irradiated mice, we observed two waves of p16 activation in the kidney area: an early phase at 3-5 months after irradiation, and a late phase at 11-13 months. These data have been included in the new Figure 3 of the revised manuscript.

Finally, we investigated *in vitro* whether supernatants from senescent endothelial cells trigger podocyte apoptosis in co-culture experiments. Results clearly showed that this was the case. The new data are shown in the new Figure 6G.

3. What was the rationale for focusing on PAI-1, IL-1b, IL-6 and MMP-13 as SASP components? Why was it surprising that VEGF-A was downregulated? The authors show that PAI-1 is upregulated in glomeruli, but can this be pinpointed to endothelial cells.

We focused on these SASP components since they have been previously shown to be involved in kidney deterioration (*Eddy AE et al, J Am Soc Nep 2006, 17:2999-3012; Lemos DR et al, J Am Soc Nep 2018, 29:1690-1705; Amdur RL et al, Clin J Am Soc Nep 2016, 11:1546-1556; Mayer G et al, Diabetes Care 2017, 40:391-397*).

We were surprised to see VEGF-A decreasing since it was reported to increase during senescence.

Concerning PAI-1 staining, to provide further evidence that it is located to endothelial cells, we performed co-immunostaining experiments with PAI-1 and griffonia in mouse kidneys and with PAI-1 and CD34 in human biopsies. We observed that PAI-1 is expressed in close vicinity to griffonia and CD34. The fact that PAI-1 is a secreted protein may account for it not perfectly overlapping with griffonia/CD34 staining. The new results are shown in the new Figure 7C and Figure EV2.

4. Similar questions outlined in points 1-3 should be addressed in the set of experiments where irradiation is used to induce senescence and glomerular lesions.

We agree with the Reviewer and have performed the same experiments in aging mice and irradiated mice.

5. A strategy is then taken to delete PAI-1 in the endothelium using VE-cadherin Cre. A key control is missing, animals with Cre alone, do the authors have data on this as Cre recombinase can itself promote glomerular injury (Balkawade Am J Physiol Renal Physiol 2019 316: F1026-40). VE-cadherin Cre will delete vessels in the whole-body of the mouse not just the kidney so was there any phenotype in other organs. One time-point is examined (at 22 weeks) so only an association between senescence and podocyte apoptosis can be made. As highlighted above the paper would be strengthened if multiple time-points had been examined.

It is true that a previous report indicated that Cre could potentially promote glomerular lesions by itself. However, we have never observed this effect in other experimental transgenic models that we generated using NPHS2-Cre (*Canaud G et al, Nat Med 2013,19:1288-1296*). Along the same line, a number of other studies published in prestigious journals (*Ding N et al, Nat Med 2006, 12:1081-1087, Welsh GI et al, Cell Metab 2010, 12:329-340, Bollee G et al, Nat Med 2011, 17:1242-1250, Henique C et al, Nat Commun 2017, doi: 10.1038s41467-017-01885-7 ….)* did not report any toxic effects of Cre. Whether differences in the genetic background can explain this difference is an interesting hypothesis. In addition, it is worth noting that in our model, mice expressing Cre are rather protected from glomerulosclerosis in

aged mice. Although it is very likely that the protection is due to PAI-1 gene inactivation, it seems that in our model, Cre expression is not toxic.

Concerning the phenotype of other organs in *PAI-1$^{\Delta endo}$* aged mice, we did not notice any macroscopic abnormalities at the time of sacrifice. As we have harvested the heart, a pathologist analyzed the impact of PAI-1 deletion on paraffin embedded tissue. The results showed that the morphological appearance was normal and comparable between *PAI-1$^{\Delta endo}$* aged mice and control littermates. We have not included these data in the revised version of the manuscript, but if the Reviewer feels that this is important, we will be happy to do so.

As indicated above, different time-points have now been studied and the results have been included in the revised version of the manuscript.

6. Cell culture is used to examine endothelial-podocyte crosstalk in more detail. Primary glomerular endothelial cells are used - can the authors show they express appropriate markers and if they are maintained as passage number increases. Can quantification of the actin cytoskeleton be performed once endothelial cell supernatants have been added to podocytes? In these experiments, 30 cells from 3 independent experiments are assessed for focal adhesions. The data is presented for each cell, but are there also differences if the means of each experiment are plotted. Is there apoptosis of the podocyte as seen in vitro?

As shown in Reviewer Figure 2, the primary glomerular endothelial cells used express classical endothelial markers, such as PECAM1, TIE1 or CDH5. More importantly, these markers were maintained from passage 9 to passage 17. We did not consider it mandatory to include these data in the revised version of the manuscript, but if the Reviewer feels that this is important, we will be happy to do so.

It is true that we assessed the number of focal adhesions in 30 cells from 3 independent experiments, and this is the reason why we decided to present the data for each cell. However, if we plot the mean of each experiment, the difference remains statistically significant (see Reviewer Figure 3).

In order to provide evidence that the supernatants from senescent endothelial cells trigger podocyte apoptosis, we performed FACS experiments using Annexin-V on podocytes incubated with supernatants from endothelial cells at early (P9) or late (P17) passage in the presence, or not, of Tiplaxtinin. Interestingly, we observed an increase in apoptosis of podocytes incubated with late passage (senescent) supernatants as compared to early passage supernatants. More importantly, the increase was prevented by incubation with Tiplaxtinin. These data are shown in the new Figure 6G.

7. The role of PAI-1 in endothelial-podocyte crosstalk in culture is examined. Do the authors know if PAI-1 secretion is increased as the number of passages the endothelial cells undertake increase? Does Tiplaxtinin reduce the levels of PAI-1 in the supernatant? The effect on podocyte apoptosis should be evaluated.

To answer to this important question, we measured PAI-1 mRNA expression in endothelial cells at passage 9 and passage 17. The results clearly showed that PAI-1 levels increased from P9 to P17.

Concerning Tiplaxtinin, as the Reviewer very likely knows, this is a compound that acts by blocking the enzymatic activity of PAI-1. Hence, to answer the Reviewer’s concern, we attempted reverse zymography on supernatants from P9 and P17 endothelial cells treated or not with Tiplaxtinin. Although we succeeded in showing that recombinant PAI-1 activity was inhibited by Tiplaxtinin, technical problems prevented us from reaching any conclusions. In fact, incubation of the gels with the supernatants from endothelial cells resulted in a strong

background (see Reviewer Figure 1). We believe that this is due to the serum contained in the supernatant.

8. PAI-1 is then examined in young and old transplant donors, with increased glomerular expression in the older donors. Can the authors pinpoint expression to the endothelium?

We thank the reviewer for raising this important point. As requested, we performed co-immunostaining between CD34 and PAI-1 in human biopsies and observed that PAI-1 is expressed in close vicinity to CD34. As mentioned above, the fact that PAI-1 is a secreted protein may account for not perfectly overlapping with CD34 staining. The new results are shown in the new Figure 7C.

9.page 14, 2nd paragraph. It seems rather strong to suggest that PAI-1 is the mediator of the detrimental crosstalk between endothelial cells and podocytes. It is likely other mechanisms are involved. Similarly, bottom paragraph of page 15, we do not know if PAI-1 is the critical mediator in this process.

As suggested, we revised the text to ensure that the role of PAI-1 in the crosstalk between endothelial cells and podocytes is not overstated.

Minor Comments:

1. page 3, 2nd paragraph. The authors give an example of changes in glomerulosclerosis in aging but the comparison is dramatic between 18-29 years old and 70+. Is there any evidence of a step wise increase in glomerulosclerosis with increasing age?

We apologize for shortening the text and the message of the paper. In fact, the authors evaluated glomerulosclerosis in 6 groups of donors from 18 to 80 years of age and showed that the percentage of glomerulosclerosis progressively increases from 18 to 80 years (see online Table 1; *Rule AD et al., Ann Intern Med 2010, 152:561-567*).

2. page 3. An article by Baker et al, 2016 is presented as an example that senescent cells are involved in glomerulosclerosis but can more detail by provided. At the moment the paper just mentions it is a very elegant model. Please expand.

As requested, we have expanded this point in the discussion of the revised version. More importantly, in order to answer a concern raised by Reviewer 2, we have started a collaboration with Dr Baker to determine whether glomerular senescent cell depletion affects podocyte survival using the *p16 INK-ATTAC* mouse model. As the Reviewer knows, this transgenic model allows the clearance of p16 positive cells after injection of AP20187. We confirmed that the severity of glomerulosclerosis was reduced in aged transgenic mice as compared to controls. More importantly, we demonstrated that the beneficial effect of glomerular senescent cell depletion was associated with an increased number of podocytes, whereas PAI-1 expression was decreased. These data are shown in the new Figure 4.

3. page 4, end of 2nd paragraph. Rather vague. Please add details of which SASP molecules play a role in the development of glomerular lesions.

It is actually difficult to define which molecules of SASP play a role in the development of glomerulosclerosis, since none of the molecules belonging to the SASP is specific of this pathway. On the other hand, it has been shown that SASP is a heterogeneous process that is cell-type and context dependent (*Birch and Gil, Gene Dev 2020, 10.1101/gad.343129.120, and Omori S et al, Cell Metab 2020, 32:814-828*). It is worth noting that many of the SASP molecules (i.e. PAI-1, IL6, TGF-β, …) have been involved in the development of glomerulosclerosis, but whether their expression reflects the role of senescence, or not, in this

pathological setting, it is not known. This issue is now better discussed in the revised version of the manuscript.

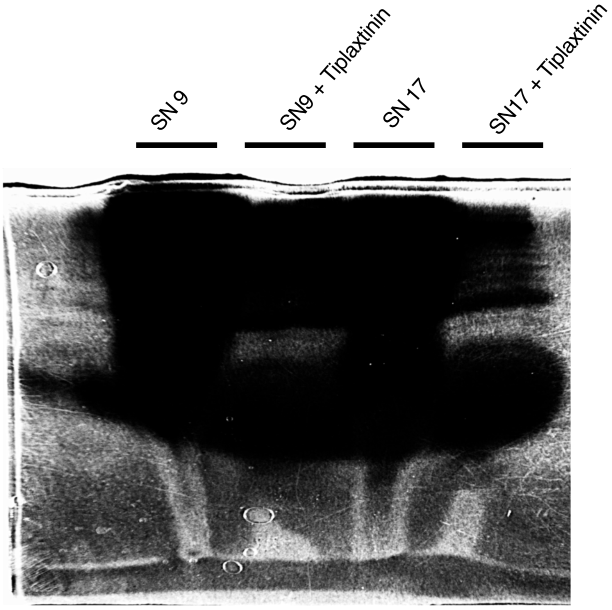

Reviewer Figure 1 : Reverse zymography of PAI-1 activity. Representative experiments of reverse zymography with supernatants from young (SN9) or senescent (SN17) endothelial cells incubated or not with Tiplaxtinin, a PAI-1 inhibitor. Zymography gel are casted with casein, plasminogen and urokinase. In the proper conditions, urokinase activates plasminogen into plasmin which will in turn digest casein. In the presence of PAI-1, urokinase cannot activate plasmin, and a band is then observed at the molecular weight of PAI-1. However, because of the huge background, very likely due to the serum, such band cannot be seen.

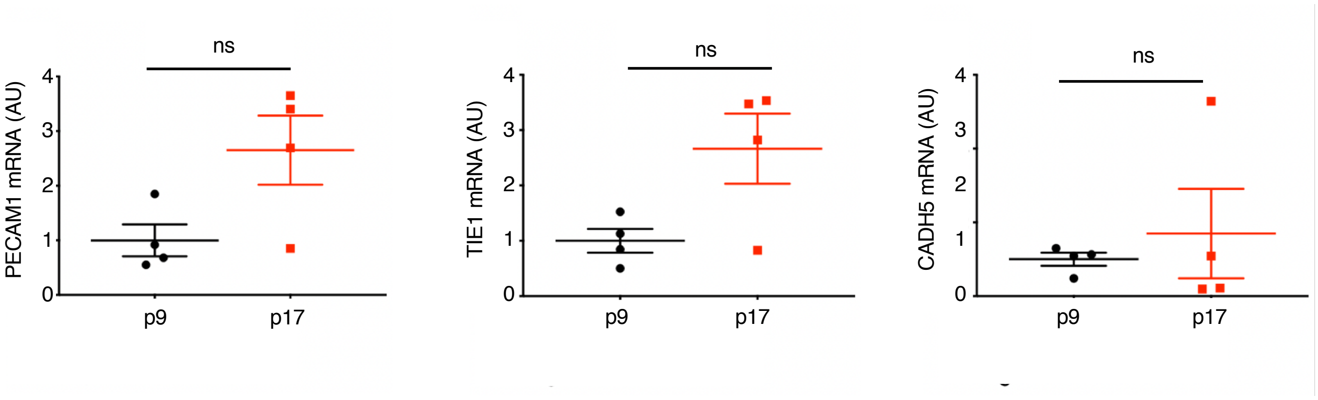

Reviewer Figure 2: Endothelial markers in GEnCs at passage 9 and 17. The expression levels of PECAM1, TIE1 and CADH5 have been evaluated by qRT-PCR. Note, that their expression is maintained throughout the culture passages.

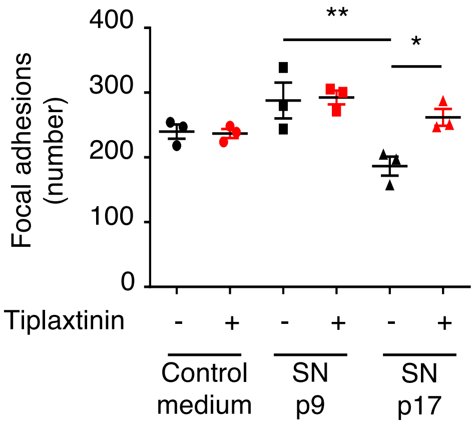

Reviewer Figure 3: Quantification of the number of focal adhesion number in podocytes after stimulation with supernatant from young (P9) or senescent (P17) GEnC, with or without Tiplaxtinin. Data are here expressed as the mean of the number of focal adhesion number per experiment (n = 3 independent experiments). Statistical analysis : * $P < 0.05$; ** $P < 0.01$.

1st Sep 2021

Dear Dr. Terzi,

Thank you for the submission of your revised manuscript to EMBO Molecular Medicine. We have now received the enclosed reports from the referees who had reviewed your original manuscript. As you will see, they are now supportive of publication, and I am therefore pleased to inform you that we will be able to accept your manuscript once the following minor points will be addressed:

1/ Referees' comments:
Please address the minor comments from referees #2 and #3 in writing or experimentally where needed.

2/ Main manuscript text:
- Please address the queries from our data editors in track changes mode in the main manuscript file labelled 'Data edited MS file'. Please use this file for any further modification (in track changes mode).
- During our standard cross-check analysis, minor text similarities with previously published material were found in the discussion (see screenshot attached), therefore please slightly modify these sentences (page 17).
- Please remove "Data not shown" (p. 19). As per our guidelines on "Unpublished Data" the journal does not permit citation of "Data not shown". All data referred to in the paper should be displayed in the main or Expanded View figures.
- Material and methods:
o Animals: please indicate the origin of the mice.
o Cells: please indicate the origin of the human immortalized podocytes. Please indicate whether cells were tested for mycoplasma contamination.
- It is mandatory to include a 'Data Availability' section after the Materials and Methods. Before submitting your revision, primary datasets produced in this study need to be deposited in an appropriate public database, and the accession numbers and database listed under 'Data Availability'.
In case you have no data that requires deposition in a public database, please state so in this section. Note that the Data Availability Section is restricted to new primary data that are part of this study.
Please fill section F/18 of the checklist accordingly.
- Author contributions: Clement Nguyen, Lucille Amrouche, Julien Le Guen are currently missing, please update.
- Please update the reference format to have them in alphabetical order and with 10 authors listed before et al.
- Please provide the funding information in the submission system.

3/ Figures and Appendix:
- Please provide in the figures or in their legends exact p values, not a range. Some people found that to keep the figures clear, providing a supplemental table with all exact p-values was preferable. You are welcome to do this if you want to.
- Please upload the figures in individual separate files.
- Please make sure that the figures are referenced in the text in the chronological order (currently Fig. EV2 is called out before Fig. EV1).
- Please add a table of content in the Appendix file, and update the nomenclature to Appendix Table S1 and Appendix Table S2.

4/ We would also encourage you to include the source data for figure panels that show essential data. Numerical data should be provided as individual .xls or .csv files (including a tab describing the data). For blots or microscopy, uncropped images should be submitted (using a zip archive if multiple images need to be supplied for one panel). Additional information on source data and instruction on how to label the files are available at
.

5/ Thank you for providing The Paper Explained section. To better fit our style and format, please shorten it a bit. You might refer to any of our published primary research articles for an example.

6/ Thank you for providing a synopsis. I added minor edits to fit our style and format. Please amend as you see fit, remove the synopsis from the main manuscript file and upload it separately (or send it via email):

Kidneys develop lesions with age, and in particular glomerulosclerosis, but the molecular mechanisms involved in the deterioration process are unclear. Here, an unexpected role for glomerular endothelial cells during aging was uncovered.
• Senescent glomerular endothelial cells increased with age, whereas the number of podocytes decreased.
• The existence of a detrimental crosstalk between senescent glomerular endothelial cells and podocyte was demonstrated in vivo and in vitro.
• Depletion of senescent cells prevented podocyte loss with age.
• PAI-1 was a critical mediator of this cross-stalk, and its selective inactivation in endothelial cell preserved kidneys from glomerulosclerosis during aging.

• PAI-1 immunostaining predicted kidney allograft dysfunction after transplantation from elderly donors. PAI-1 excretion was increased in the urine of elderly patients with recognized aging nephropathy.

Thank you for providing a nice synopsis image. We have resized it. Please let us know if you agree with the synopsis and eTOC attached. Please note that these would be the final versions and changes during proofing are usually not allowed.

7/ As part of the EMBO Publications transparent editorial process initiative, EMBO Molecular Medicine will publish online a Review Process File (RPF) to accompany accepted manuscripts.
This file will be published in conjunction with your paper and will include the anonymous referee reports, your point-by-point response and all pertinent correspondence relating to the manuscript. Let us know whether you agree with the publication of the RPF and as here, IF YOU WANT TO REMOVE OR NOT any figures from it prior to publication.
Please note that the Authors checklist will be published at the end of the RPF.

I look forward to receiving your revised manuscript.

Yours sincerely,

Lise Roth

Lise Roth, PhD
Editor
EMBO Molecular Medicine

***** Reviewer's comments *****

Referee #1 (Remarks for Author):

The authors have properly addressed the issues that I have previously raised. I am now supporting the publication of this revised version.

Referee #2 (Comments on Novelty/Model System for Author):

The experiments are well designed, performed and analysed, and the authors use state-of-the-art models to address the relevant questions, including genetically-engineered mouse models to manipulate PAI-1 and senescence, aging and irradiation, and also human samples. The role of senescence is a hot topic in research and how it associates with age-related diseases, including kidney disease. Also, there is a clear translational component in this research.

Referee #2 (Remarks for Author):

This is now the revised version of the manuscript submitted by Cohen et al. The authors have addressed most of my original concerns, in particular:

1. The authors have provided a more robust assessment of cellular senescence in glomerular endothelial cells.
2. The new in vivo experiment performed with p16 INK-ATTAC mice provide evidence of a causal link between p16-positive glomerular senescent cells, the accumulation of PAI-1 and decreased podocytes numbers. I would suggest that the authors add here (perhaps as a supplementary figure) an internal control showing that AP+ treatment of p16 INK-ATTAC mice results in reduced p16-positive cell numbers in the glomeruli, if possible. I guess the group of Baker did that control.
3. The authors show further mechanistic insights on the crosstalk between senescent endothelial cells and podocytes in Figure 6G.

4. Further evidence of the association of p16+ areas and PAI-1 in the glomeruli, suggesting a connection between the induction of endothelial senescence and PAI-1 secretion.

Altogether, the manuscript has been significantly improved, and I do not have further requests or concerns at this stage. I congratulate the authors for the work done and for, in my view, such important contribution to the fields of senescence and kidney disease

Referee #3 (Remarks for Author):

Cohen and colleagues present a revised version of their manuscript examining the crosstalk between senescence endothelial cells and podocytes in the development of glomerular lesions during aging. The authors have been responsive to the comments of the reviewer with additional data including double immunostaining to pinpoint the identity of senescence cells, time-course of the pathology in aging mice, exploration of mice expressing luciferase under the control of the p16 promoter, investigating whether supernatants from senescence endothelial cells trigger podocyte apoptosis and improvements in the text. Collectively, these changes have significantly enhanced the manuscript. There are a few minor queries remaining highlighted below.

1. In Figure 3C-D, new data is presented using bioluminescence to trace senescence cells in mice expressing luciferase under the control of the p16 promoter. The authors conclude that the data in Figure 3D shows two-waves of p16 activation, but the data doesn't seem that convincing with lots of variability in the earlier time-points. The authors should consider refining this conclusion. Why are the values so much lower than Figure 3C?

2. On page 12. The authors show decreased PAI-1 expression in p16 INK-ATTAC and conclude that this suggests that senescent cells are the site of PAI-1 synthesis during aging. This seems strong - this change could reflect the fact that there is reduced disease severity in these mice as seen in Figure 4A and B.

3. There are some occasions where the data is not accurately represented in the text. In Figure C, p16 and p21 are not both increased in cells between passage 9 to 17. In Figure 6G, Tiplaxtinin does not significantly prevent podocyte apoptosis (it is close). This should be refined in the text.

Ref: EMM-2021-14146-V3

Point-by-point responses to reviewer's comments

We thank the three Reviewers for their thoughtful comments throughout all the reviewing process. We feel that the revised version of the manuscript has been considerably improved by their inputs/criticisms.

Referee #2

(Comments on Novelty/Model System for Author):

The experiments are well designed, performed and analysed, and the authors use state-of-the-art models to address the relevant questions, including genetically-engineered mouse models to manipulate PAI-1 and senescence, aging and irradiation, and also human samples. The role of senescence is a hot topic in research and how it associates with age-related diseases, including kidney disease. Also, there is a clear translational component in this research.

(Remarks for Author):

This is now the revised version of the manuscript submitted by Cohen et al. The authors have addressed most of my original concerns, in particular:

1. The authors have provided a more robust assessment of cellular senescence in glomerular endothelial cells.

2. The new in vivo experiment performed with p16 INK-ATTAC mice provide evidence of a causal link between p16-positive glomerular senescent cells, the accumulation of PAI-1 and decreased podocytes numbers. I would suggest that the authors add here (perhaps as a supplementary figure) an internal control showing that AP+ treatment of p16 INK-ATTAC mice results in reduced p16-positive cell numbers in the glomeruli, if possible. I guess the group of Baker did that control.

3. The authors show further mechanistic insights on the crosstalk between senescent endothelial cells and podocytes in Figure 6G.

4. Further evidence of the association of p16+ areas and PAI-1 in the glomeruli, suggesting a connection between the induction of endothelial senescence and PAI-1 secretion.

Altogether, the manuscript has been significantly improved, and I do not have further requests or concerns at this stage. I congratulate the authors for the work done and for, in my view, such important contribution to the fields of senescence and kidney disease.

We are glad to see that the Reviewer recognizes that we have addressed the concerns he/she has raised in the first revision of the manuscript.

As far as the possibility to determine whether AP treatment results in reduced p16-positive cells in the glomeruli of p16 INK-ATTAC mice, unfortunately, both our Lab and Baker's Lab failed to set up experimental conditions able to detect p16 in mouse tissue. Thus, this experiment could not be performed.

Referee #3 (Remarks for Author):

Cohen and colleagues present a revised version of their manuscript examining the crosstalk between senescence endothelial cells and podocytes in the development of glomerular lesions during aging. The authors have been responsive to the comments of the reviewer with additional data including double immunostaining to pinpoint the identity of senescence cells, time-course of the pathology in aging mice, exploration of mice expressing luciferase under the control of the p16 promoter, investigating whether supernatants from senescence endothelial cells trigger podocyte apoptosis and improvements in the text. Collectively, these changes have significantly enhanced the manuscript. There are a few minor queries remaining highlighted below.

We are glad to see that the Reviewer recognizes that we have addressed the concerns he/she has raised in the first revision of the manuscript.

1. In Figure 3C-D, new data is presented using bioluminescence to trace senescence cells in mice expressing luciferase under the control of the p16 promoter. The authors conclude that the data in Figure 3D shows two-waves of p16 activation, but the data doesn't seem that convincing with lots of variability in the earlier time-points. The authors should consider refining this conclusion. Why are the values so much lower than Figure 3C?

We thank the Reviewer for raising this important point. We agree that the results of bioluminescence (BLI) are quite variable. However, there is no doubt that BLI start to increase from 18 months of age and is uncontestably higher in kidneys of 24-months old mice as compared to kidneys of 6-months old mice (Figure 3C). Because of the variability, the differences are less striking in Figure 3D. Hence, as requested, the conclusion has been refined.

The fact that the values are lower in Figure 3D compared to Figure 3C is explained by the way we expressed the data. In Figure 3C, the data are expressed as the intensity of BLI over body weight, as the experiment lasted for almost 2 years, implying a change of mice morphology. On the other hand, in Figure 3D, the results are expressed as the ratio of kidney area BLI over total body BLI, limiting the background effect. We chose this method for Figure 3D since mice were subjected to total body irradiation, which potentially led to p16 activation in all the tissues. This is explained in the Method section.

2. On page 12. The authors show decreased PAI-1 expression in p16 INK-ATTAC and conclude that this suggests that senescent cells are the site of PAI-1 synthesis during aging. This seems strong - this change could reflect the fact that there is reduced disease severity in these mice as seen in Figure 4A and B.

We agree with the Reviewer; thus, we modified the text accordingly.

3. There are some occasions where the data is not accurately represented in the text. In Figure C, p16 and p21 are not both increased in cells between passage 9 to 17. In Figure 6G, Tiplaxtinin does not significantly prevent podocyte apoptosis (it is close). This should be refined in the text.

We thank the Reviewer for raising this point. As requested, we have now better checked the text and insured that all the data have been correctly described in the Result section.

22nd Sep 2021

Dear Dr. Terzi,

We are pleased to inform you that your manuscript is accepted for publication and is now being sent to our publisher to be included in the next available issue of EMBO Molecular Medicine!

We would like to remind you that as part of the EMBO Publications transparent editorial process initiative, EMBO Molecular Medicine will publish a Review Process File online to accompany accepted manuscripts. If you do NOT want the file to be published or would like to exclude figures, please immediately inform the editorial office via e-mail.

Congratulations on your interesting work!

With kind regards,

Lise Roth

Lise Roth, Ph.D
Editor
EMBO Molecular Medicine

Follow us on Twitter @EmboMolMed
Sign up

EMBO PRESS

YOU MUST COMPLETE ALL CELLS WITH A PINK BACKGROUND ↓

PLEASE NOTE THAT THIS CHECKLIST WILL BE PUBLISHED ALONGSIDE YOUR PAPER

Corresponding Author Name: Fabiola Terzi
Journal Submitted to: EMBO Molecular Medicine
Manuscript Number: EMM-2021-14146

USEFUL LINKS FOR COMPLETING THIS FORM

http://www.antibodypedia.com
http://1degreebio.org
http://www.equator-network.org/reporting-guidelines/improving-bioscience-research-repor
http://grants.nih.gov/grants/olaw/olaw.htm
http://www.mrc.ac.uk/Ourresearch/Ethicsresearchguidance/Useofanimals/index.htm
http://ClinicalTrials.gov
http://www.consort-statement.org
http://www.consort-statement.org/checklists/view/32-consort/66-title
http://www.equator-network.org/reporting-guidelines/reporting-recommendations-for-tumo
http://datadryad.org
http://figshare.com
http://www.ncbi.nlm.nih.gov/gap
http://www.ebi.ac.uk/ega
http://biomodels.net/
http://biomodels.net/miriam/
http://jjj.biochem.sun.ac.za
http://oba.od.nih.gov/biosecurity/biosecurity_documents.html
http://www.selectagents.gov/

Reporting Checklist For Life Sciences Articles (Rev. June 2017)

This checklist is used to ensure good reporting standards and to improve the reproducibility of published results. These guidelines are consistent with the Principles and Guidelines for Reporting Preclinical Research issued by the NIH in 2014. Please follow the journal's authorship guidelines in preparing your manuscript.

A- Figures
1. Data
The data shown in figures should satisfy the following conditions:

- the data were obtained and processed according to the field's best practice and are presented to reflect the results of the experiments in an accurate and unbiased manner.
- figure panels include only data points, measurements or observations that can be compared to each other in a scientifically meaningful way.
- graphs include clearly labeled error bars for independent experiments and sample sizes. Unless justified, error bars should not be shown for technical replicates.
- if n< 5, the individual data points from each experiment should be plotted and any statistical test employed should be justified
- Source Data should be included to report the data underlying graphs. Please follow the guidelines set out in the author ship guidelines on Data Presentation.

2. Captions
Each figure caption should contain the following information, for each panel where they are relevant:

- a specification of the experimental system investigated (eg cell line, species name).
- the assay(s) and method(s) used to carry out the reported observations and measurements
- an explicit mention of the biological and chemical entity(ies) that are being measured.
- an explicit mention of the biological and chemical entity(ies) that are altered/varied/perturbed in a controlled manner.

- the exact sample size (n) for each experimental group/condition, given as a number, not a range;
- a description of the sample collection allowing the reader to understand whether the samples represent technical or biological replicates (including how many animals, litters, cultures, etc.).
- a statement of how many times the experiment shown was independently replicated in the laboratory.
- definitions of statistical methods and measures:
  - common tests, such as t-test (please specify whether paired vs. unpaired), simple χ2 tests, Wilcoxon and Mann-Whitney tests, can be unambiguously identified by name only, but more complex techniques should be described in the methods section;
  - are tests one-sided or two-sided?
  - are there adjustments for multiple comparisons?
  - exact statistical test results, e.g., P values = x but not P values < x;
  - definition of 'center values' as median or average;
  - definition of error bars as s.d. or s.e.m.

Any descriptions too long for the figure legend should be included in the methods section and/or with the source data.

In the pink boxes below, please ensure that the answers to the following questions are reported in the manuscript itself. Every question should be answered. If the question is not relevant to your research, please write NA (non applicable). We encourage you to include a specific subsection in the methods section for statistics, reagents, animal models and human subjects.

B- Statistics and general methods

Please fill out these boxes ↓ (Do not worry if you cannot see all your text once you press return)

1.a. How was the sample size chosen to ensure adequate power to detect a pre-specified effect size?	No specific statistical test was used to chose the sample size for animal experiments. Fortunately, several of these animals were used in other studies, limiting the number of specifically bred animals for our study.
1.b. For animal studies, include a statement about sample size estimate even if no statistical methods were used.	For animal sample size estimate, the minimum number of animals to obtain a statistical effect was chosen.
2. Describe inclusion/exclusion criteria if samples or animals were excluded from the analysis. Were the criteria pre-established?	No animal was excluded from the analysis.
3. Were any steps taken to minimize the effects of subjective bias when allocating animals/samples to treatment (e.g. randomization procedure)? If yes, please describe.	N/A
For animal studies, include a statement about randomization even if no randomization was used.	No randomization was used
4.a. Were any steps taken to minimize the effects of subjective bias during group allocation or/and when assessing results (e.g. blinding of the investigator)? If yes please describe.	All quantifications of histopathological or immunohistochemical studies were performed blindly by the investigator
4.b. For animal studies, include a statement about blinding even if no blinding was done	Blinding of the investigator about the group of animals was performed as often as possible.
5. For every figure, are statistical tests justified as appropriate?	Yes
Do the data meet the assumptions of the tests (e.g., normal distribution)? Describe any methods used to assess it.	Yes. For example, for quantitative data comparison, we tested the normality of the distribution by performing a Shapiro test. If the distribution was normal, a t-test was performed. Otherwise, a Mann-Whitney test was performed.
Is there an estimate of variation within each group of data?	Yes
Is the variance similar between the groups that are being statistically compared?	Yes

C- Reagents

6. To show that antibodies were profiled for use in the system under study (assay and species), provide a citation, catalog number and/or clone number, supplementary information or reference to an antibody validation profile. e.g., Antibodypedia (see link list at top right), 1DegreeBio (see link list at top right).	mouse monoclonal anti-p21 (BD Bioscience, ref 556431) at 1/200 (Deng et al. Nat Communications 2020), rat monoclonal anti-CD34 (eBiosciences, ref 14-0341-85) at 1/50 (Yang Z, et al. Role of CD34 family members in lumen formation in the developing kidney. Dev Biol. 2016, rabbit polyclonal anti-53BP1 (Abcam, ref ab36823) at 1/100 (Simon M et al. LINE1 Derepression in Aged Wild-Type and SIRT6-Deficient Mice Drives Inflammation. Cell Metab 29:871-885.e5 (2019), mouse monoclonal anti-WT1 (Wilms Tumor 1) (Dako, ref M3561) at 1/50 (Zhao L et al. A piggyBac transposon- and gateway-enhanced system for efficient BAC transgenesis. Dev Dyn. 2014;243:1086-94 , rabbit polyclonal anti-PAI-1 (Abcam, ref ab28207) at 1/100 (Bennett RE et al. Tau induces blood vessel abnormalities and angiogenesis-related gene expression in P301L transgenic mice and human Alzheimer's disease. Proc Natl Acad Sci U S A 115:E1289-E1298 (2018)). mouse monoclonal anti-CD34 (Dako, ref M7165) at 1/100 (Fina L, et al. Blood 1990); monoclonal guinea-pig anti-nephrin (Progen, ref GP-N2) at 1/100 (Bussolati et al. JASN 2005); monoclonal rabbit anti-pH2AX (Cell Signaling, ref 9718S) at 1/200 (Abe et al. Diagnostic cytopathology 2012); griffonia simplicifolia-FITC (Vector, ref FL-1101) at 1/50 (Al-Darraji et al. Sci Rep 2020)
7. Identify the source of cell lines and report if they were recently authenticated (e.g., by STR profiling) and tested for mycoplasma contamination.	Human immortalized podocytes were cultured as previously described (Saleem et al, 2002). Human primary glomerular endothelial cells (GEnC) were purchased from Cell System (ACBRI 128) and cultured according to manufacturer's instructions. Mycoplasma test was negative.

* for all hyperlinks, please see the table at the top right of the document

D- Animal Models

8. Report species, strain, gender, age of animals and genetic modification status where applicable. Please detail housing and husbandry conditions and the source of animals.	Mice used for experiments were male and female C57Bl/6, PAI-1flox / flox, VeCadh-Cre+/- (Rannou et al, 2015; Alva et al, 2006), p16-INK-ATTAC mice, p16+/luc mice (Soysouvanh et al. 2020). PAI-1flox / flox mice were crossed with VeCadh-Cre+/- to generate PAI-1Δendo mice, in which PAI-1 (Serpine 1 gene) was selectively deleted in endothelial cells. p16 INK-ATTAC mice were housed and sacrificed in D.J Baker lab in Mayo clinic, Rochester, MN, USA. Mice were fed ad libitum and housed at constant ambient temperature in a 12-h light, 12-h dark cycle.
9. For experiments involving live vertebrates, include a statement of compliance with ethical regulations and identify the committee(s) approving the experiments.	Animal procedures were approved by the "Services Vétérinaires de la Préfecture de Police de Paris", by the "Ministère de l'Enseignement Supérieur de la Recherche et de l'Innovation" and by the ethical committee of the Paris Descartes University
10. We recommend consulting the ARRIVE guidelines (see link list at top right) (PLoS Biol. 8(6), e1000412, 2010) to ensure that other relevant aspects of animal studies are adequately reported. See author guidelines, under 'Reporting Guidelines'. See also: NIH (see link list at top right) and MRC (see link list at top right) recommendations. Please confirm compliance.	We confirm compliance to the ARRIVE guidelines.

E- Human Subjects

11. Identify the committee(s) approving the study protocol.	CPP Ile de France XI ; referencence 11017
12. Include a statement confirming that informed consent was obtained from all subjects and that the experiments conformed to the principles set out in the WMA Declaration of Helsinki and the Department of Health and Human Services Belmont Report.	The protocol was approved by the Institutional Review Board of Necker Hospital; informed written consent was obtained from each patient. The experiment conformed to the principles set out in the WMA Declaration of Helsinki and the Department of Health and Human Services Belmont Report.
13. For publication of patient photos, include a statement confirming that consent to publish was obtained.	N/A
14. Report any restrictions on the availability (and/or on the use) of human data or samples.	No restrictions
15. Report the clinical trial registration number (at ClinicalTrials.gov or equivalent), where applicable.	N/A
16. For phase II and III randomized controlled trials, please refer to the CONSORT flow diagram (see link list at top right) and submit the CONSORT checklist (see link list at top right) with your submission. See author guidelines, under 'Reporting Guidelines'. Please confirm you have submitted this list.	N/A
17. For tumor marker prognostic studies, we recommend that you follow the REMARK reporting guidelines (see link list at top right). See author guidelines, under 'Reporting Guidelines'. Please confirm you have followed these guidelines.	N/A

F- Data Accessibility

18: Provide a "Data Availability" section at the end of the Materials & Methods, listing the accession codes for data generated in this study and deposited in a public database (e.g. RNA-Seq data: Gene Expression Omnibus GSE39462, Proteomics data: PRIDE PXD000208 etc.) Please refer to our author guidelines for 'Data Deposition'.<br><br>Data deposition in a public repository is mandatory for:<br>a. Protein, DNA and RNA sequences<br>b. Macromolecular structures<br>c. Crystallographic data for small molecules<br>d. Functional genomics data<br>e. Proteomics and molecular interactions	We did no provide any data that require deposition in a public database
19. Deposition is strongly recommended for any datasets that are central and integral to the study; please consider the journal's data policy. If no structured public repository exists for a given data type, we encourage the provision of datasets in the manuscript as a Supplementary Document (see author guidelines under 'Expanded View' or in unstructured repositories such as Dryad (see link list at top right) or Figshare (see link list at top right).	N/A
20. Access to human clinical and genomic datasets should be provided with as few restrictions as possible while respecting ethical obligations to the patients and relevant medical and legal issues. If practically possible and compatible with the individual consent agreement used in the study, such data should be deposited in one of the major public access-controlled repositories such as dbGAP (see link list at top right) or EGA (see link list at top right).	N/A
21. Computational models that are central and integral to a study should be shared without restrictions and provided in a machine-readable form. The relevant accession numbers or links should be provided. When possible, standardized format (SBML, CellML) should be used instead of scripts (e.g. MATLAB). Authors are strongly encouraged to follow the MIRIAM guidelines (see link list at top right) and deposit their model in a public database such as Biomodels (see link list at top right) or JWS Online (see link list at top right). If computer source code is provided with the paper, it should be deposited in a public repository or included in supplementary information.	N/A

G- Dual use research of concern

22. Could your study fall under dual use research restrictions? Please check biosecurity documents (see link list at top right) and list of select agents and toxins (APHIS/CDC) (see link list at top right). According to our biosecurity guidelines, provide a statement only if it could.	No
